# Angiocrine extracellular vesicles impose mesenchymal reprogramming upon proneural glioma stem cells

**DOI:** 10.1038/s41467-022-33235-7

**Published:** 2022-09-19

**Authors:** Lata Adnani, Jordan Kassouf, Brian Meehan, Cristiana Spinelli, Nadim Tawil, Ichiro Nakano, Janusz Rak

**Affiliations:** 1 Research Institute of the McGill University Health Centre, Montreal, QC H4A 3J1 Canada; 2 Department of Biochemistry, McGill University, Montreal, QC H4A 3J1 Canada; 3 Department of Neurosurgery, Hokuto Social Medical Corporation, Hokuto Hospital, Kisen-7-5 Inadacho, Obihiro, Hokkaido 080-0833 Japan; 4 Department of Pediatrics, McGill University, Montreal, QC H4A 3J1 Canada

**Keywords:** Cancer stem cells, Cancer stem cells, CNS cancer, Cancer stem cells, Tumour angiogenesis

## Abstract

Glioblastoma (GBM) is an incurable form of primary astrocytic brain tumor driven by glioma stem cell (GSC) compartment closely associated with the vascular niche. GSC phenotypes are heterogeneous and range from proneural to mesenchymal-like, the latter characterised by greater invasiveness. Here we document the secretory (angiocrine) role of endothelial cells and their derived extracellular vesicles (EVs) as drivers of proneural-to-mesenchymal reprogramming of GSCs. These changes involve activation of matrix metalloproteinases (MMPs) and NFκB, and inactivation of NOTCH, while altering responsiveness to chemotherapy and driving infiltrative growth in the brain. Our findings suggest that EV-mediated angiocrine interactions impact the nature of cellular stemness in GBM with implications for disease biology and therapy.

## Abstract (web summary)

Glioma stem-like cells (GSCs) exhibit plasticity during proneural-to-mesenchymal transition. Here the authors show that extracellular vesicles from endothelial cells can promote this transition of GSCs through activation of MMPs and NFkB, and inactivation of NOTCH.

## Introduction

Glioblastoma multiforme (GBM) is an aggressive and incurable brain tumor with pronounced vascularity. GBM is thought to arise from glioma stem-like cells (GSCs) of which at least two subtypes have been identified and described as either proneural (PN) or mesenchymal (MES)^1–6^. The gene expression profiles of GSCs are reminiscent of the corresponding GBM subgroups^7^ and imply numerical preponderance^8^. Notably, patients with mesenchymal GBM tend to have a poorer prognosis relative to patients with proneural GBM^7^ (Supplementary Fig. 1a). Some of the distinctive features of proneural GSCs include the expression of SOX2, NES, and NOTCH1, while mesenchymal GSCs are enriched for CD44, VIM, and EGFR along with activation of the NFκB and glycolysis metabolism pathways^9,10^ (Supplementary Fig. 1b–h). These phenotypes exhibit a degree of plasticity with proneural-to-mesenchymal transition (PMT) often associated with relapse, increased invasiveness and radiation resistance^11–15^. PMT is characterized by upregulation of CD44, BCL2A^3^, and NFκB^10,15^ on the background of proneural GSCs.

Interactions with the brain vasculature represent a hallmark of both neural stem cells and GSCs, epitomized by formation of endothelial stem cell niches^16–18^. Interrelationships between GSCs and endothelial cells have also emerged in the context of angiogenesis^19,20^ and other processes driving tumor neovascularization^21–23^, with ongoing identification of their respective mediators^16,17^. On the other hand, endothelium is regarded as a secretory tissue, the paracrine (angiocrine) effects of which are thought to be mediated by either soluble proteins, or multimolecular carriers, such as extracellular vesicles (EVs) including exosomes^24^. Indeed, the endothelial secretome has recently emerged as a potent regulatory mechanism for cancer cells, their niches, and across tumor microenvironment^25–27^. However, the present understanding of the nature and scope of angiocrine influences on different subsets of cancer cells^25^, stem cells^28^ and GSCs remains relatively limited^16^.

Here, we show that endothelial cell derived EVs (EEVs) not only harbor the ability to modify GSC behavior, but also potentiate a switch in GSC subtype. Specifically, the conditioned medium derived from endothelial cells prevents sphere formation, reduces proneural hallmarks, induces mesenchymal hallmarks, causes increased migration of proneural GSCs, and facilitates increased tumorigenicity in mice xenografted with proneural GSCs pre-treated with endothelial cell derived conditioned media relative to the controls. These findings are in harmony with the results obtained when proneural GSCs were treated with endothelial cell derived EVs (EEVs). We have identified MMPs as a key cargo component of EEVs, which when taken up by proneural cells inaugurates the mesenchymal signaling pathway, NFκB. This study sheds new light on the alteration of proneural GSCs not after an intervention, such as surgical resection, radiation or chemotherapy, but during the development of a naïve primary GBM. This change entails EEV uptake by GSCs which leads to the downregulation of the NOTCH pathway and an upregulation of NFκB.

## Results

### Proximity between glioma stem cells and endothelium leads to stem cell sphere disruption

**Fig. 1:**
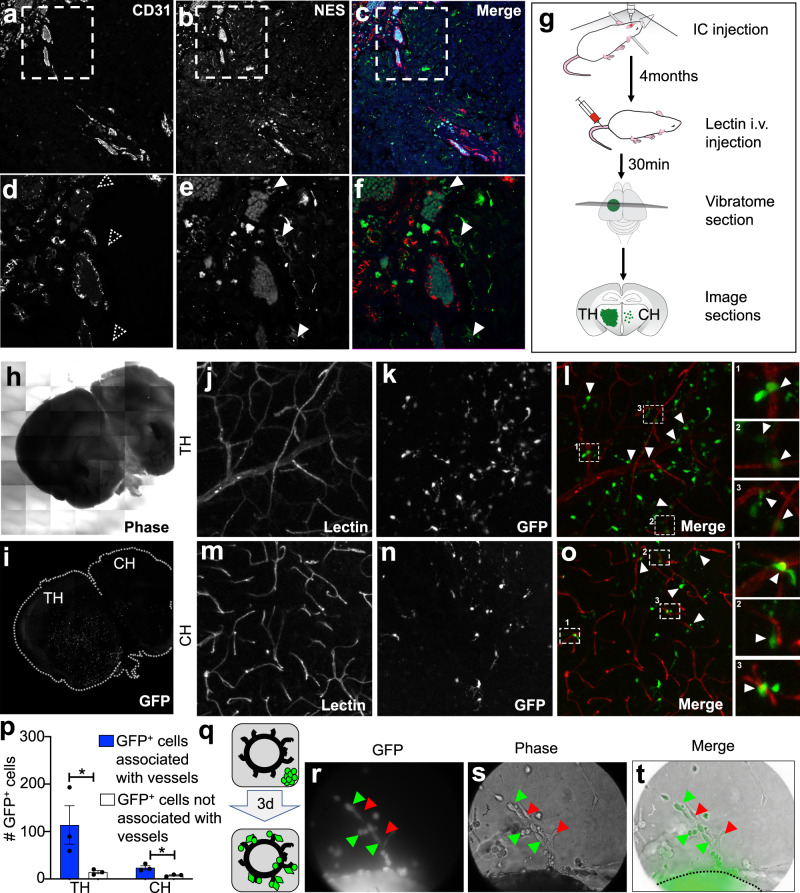
Dynamic proximity between glioma stem cells and endothelium. **a**–**f** Human GBM tissue co-stained for the endothelial cell (EC) marker, CD31, and for GSC marker, NES. White arrowheads point to NES^+^ GSCs juxta-positioned along CD31^+^ blood vessels in the GBM tissue. **g** Schematic of steps taken to obtain live mouse tissues for high resolution confocal imaging of GSCs and ECs in mouse tumor xenografts. Phase tile image (**h**) and Green fluorescence tile image (**i**) of a vibratome cut 200 µm thick coronal section of mouse brain injected with GSC157 proneural-GSCs. *Lycopersicon* lectin-stained blood vessels in the tumor hemisphere (TH; **j**) and contralateral hemisphere (CH; **m**) of the mouse tumor xenografts. GFP labeled GSC157 cells in the tumor hemisphere (**k**) and contralateral hemisphere (**n**) of the mouse brain. Merged *Lycopersicon* lectin /GFP with insets showing GSC proximity with ECs and cellular morphologies of GFP^+^ cells in the tumor hemisphere (**l**) and contralateral hemisphere (**o**). **p** Quantification of the number of GFP^+^ proneural glioma stem cells found in close proximity to-, or not near the vicinity of endothelial cells. **q** Cartoon representation of the use of mouse aortae to generate sprouting endothelial-tumor cell ‘dynamic’ co-cultures. Green cells represent GFP^+^ GSC83 that were found to migrate towards sprouting ECs. **q**–**t** Three days after GFP^+^ GSCs were placed with aortic ring, GFP^+^ GSCs migrated towards ECs and adopted more elongated cellular morphologies. Green arrowheads indicate the presence of GFP^+^ GSCs and red arrowheads point towards ECs. GSC glioma stem cells, ECs endothelial cells, TH tumor hemisphere, CH contralateral hemisphere, EC endothelial cells.

The responses of proneural and mesenchymal GSCs to endothelium-related factors have not yet been studied despite the reported proximity of these cells to the tumor vasculature^16^. Indeed, co-staining of human GBM sections for NES (marker enriched in proneural GSCs) and CD31 (endothelial cell marker) enforces such a spatial association (Fig. 1a–f). The degree of this spatial association is within the estimated diffusion limit of paracrine factors^8,29^ raising questions regarding the biological consequences it entails. We also observed that GFP-labeled human GSCs infiltrated the brain parenchyma of mice lodging either near, or away from the vascular channels, as visualised by perfusion with fluorescent *Lycopersicon* lectin (Fig. 1g–p). Interestingly, the GFP^+^ glioma stem cells closer to the vasculature assumed a more elongated, mesenchymal-like morphology (Fig. 1- insets). Moreover, a direct tropism of GSCs toward activated endothelial cells was observed in co-cultures of sprouting mouse aortic rings and GSCs (Fig. 1q–t). These findings reinforce a potential for the various degrees of proximity to enable a paracrine endothelial-GSC interaction.

**Fig. 2:**
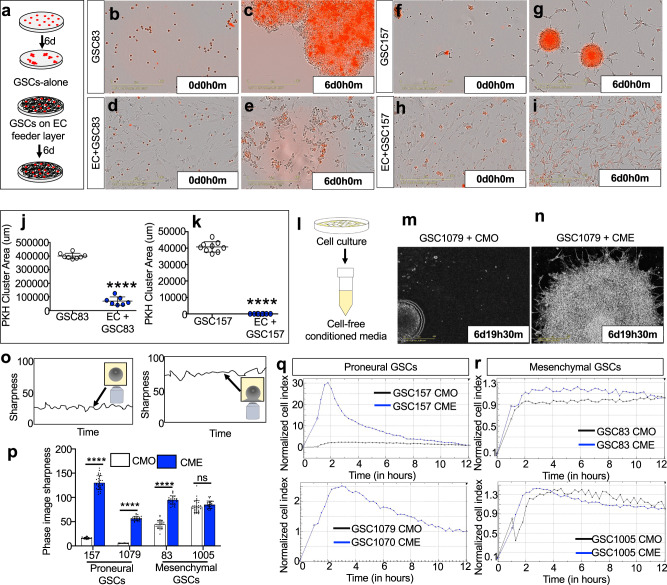
Paracrine disruption of glioma stem cell sphere formation by endothelial cells. **a** Schematic of culturing GSCs alone, or in co-culture with HUVECs for 6 days. Red represents GSCs and light green represents HUVECs. **b**, **c** Mesenchymal GSC83 stained with PKH26 (red) were cultured alone for 6 days and growth patterns were recorded using Incucyte imaging. **d**, **e** Mesenchymal GSC83 stained with PKH26 and co-cultured with unlabeled HUVECs for 6 days and recorded using Incucyte. **f**, **g** Proneural GSC157 stained with PKH26 and cultured for 6 days (Incucyte). **h**, **i** Proneural GSC157 stained with PKH26 and cultured with unlabeled HUVECs for 6 days (Incucyte). Incucyte quantification of PKH26 cluster area of mesenchymal GSC83 (**j**) or proneural GSC157 (**k**) cultured with or without HUVECs (day 6). **l** Schematic of obtaining conditioned media from the indicated cells lines. Conditioned media effect on proneural GSC1079 cells cultured, as spheres, in either (**m**) their own conditioned media (CMO) or in (**n**) endothelial conditioned media (CME) for 7 days. **o** Schematic of measuring cell sharpness. The cells which float in media are recorded with a smaller cell sharpness index relative to the cells which adhere to the bottom of the plate. The readings were collected using an inverted microscope of the Incucyte system. **p** Quantification of cell sharpness on day 7 for two proneural (GSC157 and GSC1079) and two mesenchymal (GSC83 and GSC1005) GSCs treated with either their own (CMO) or HBEC5i-CM (CME). Impedance-related cell shape index measured over time using xCelligence system in cultures of either (**q**) proneural (GSC157 and GSC1079) or (**r**) mesenchymal (GSC83 and GSC1005) cells treated with CMO (black line) or HBEC5i-CM (CME; blue line) for 12 h. While cell indices are dramatically impacted by CME in proneural GSC cultures, they were similar between CMO and CME conditions for mesenchymal GSCs. GSC glioma stem cells, CMO own conditioned media, CME conditioned media derived from endothelial cells, HBEC5i immortalized human brain endothelial cells, CM conditioned media.

In order to glean more insights as to the possible functional consequences of such paracrine influences, endothelial cells were cocultured with GSCs (which grow as spheres). The ability to form tumor spheres in serum free media represents a defining feature of GSCs, which is often used to interrogate their clonogenic and tumor initiating potentials^1^, while also differentiating between proneural-GSCs (tight spheres) and mesenchymal-GSCs (loose spheres) in culture. Thus, sphere cultures of several mesenchymal-GSCs (GSC83 and GSC1005) and proneural-GSCs (GSC157 and GSC1079) were incubated in the presence of endothelial cells, including brain endothelial cell line (HBEC5i; Supplementary Fig. 2), or primary endothelial cells (HUVEC; Fig. 2a–k) for 1-6 days. Remarkably, in the presence of unlabeled endothelial cells, PKH26-labeled GSCs spheres either failed to form, or rapidly disintegrated and the remaining tumor cells assumed a scattered, non-stem-cell-like, adherent and elongated morphology (Fig. 2a–k).

Next, we asked whether the effect of endothelial cells on disruption of GSC spheres (‘de-sphering’) is contact-dependent or paracrine in nature. To address this question proneural-GSC1079 spheres were incubated in conditioned media derived either from endothelial cells (CME) or their own sphere culture (CMO) for 7 days, while their responses were continuously monitored using Incucyte imaging (Fig. 2l–n; Supplementary Movies 1 & 2; Supplementary Fig. 3a, b). While CMO preserved the sphere forming capability of proneural GSCs, treatment with CME allowed the cells to gradually contact the plate and migrate out of the sphere causing it to disintegrate, thereby recapitulating the effects of endothelial cell co-culture. Interestingly, CME from several different endothelial cell populations yielded similar GSC sphere disruption effects (Supplementary Figs. 4 and 5). This was reflected in the measurements of cell sharpness (Incucyte), which captures an increase in image contrast and texture when the cells adhere to the solid substratum relative to their three-dimensional growth pattern (Fig. 2o). As expected, cell sharpness increased when proneural and mesenchymal GSCs were treated with CME relative to CMO (Fig. 2p; Supplementary Figs. 4i, j, 5i, j). Notably, these influences of CME were cell subtype specific, as sphere disruption was more dramatic in the case of proneural GSCs relative to mesenchymal GSCs, and failed to occur in cultures of pediatric high-grade glioma cells known to exhibit a distinctive biology^24^ (Supplementary Fig. 3c–h). These differentials were objectively validated using impedance measurements (xCelligence), which indicated that profiles of proneural GSCs (Fig. 2q) differed markedly from those of mesenchymal GSCs in the presence of CME (Fig. 2r). These findings imply that proneural GSCs are unable to grow and maintain tight spheres in the presence of CME suggesting that there might be differences in the clonogenicity in GSCs after CME treatment.

### Reprograming of proneural glioma stem cells into a more mesenchymal state by endothelial cell derived conditioned media

**Fig. 3:**
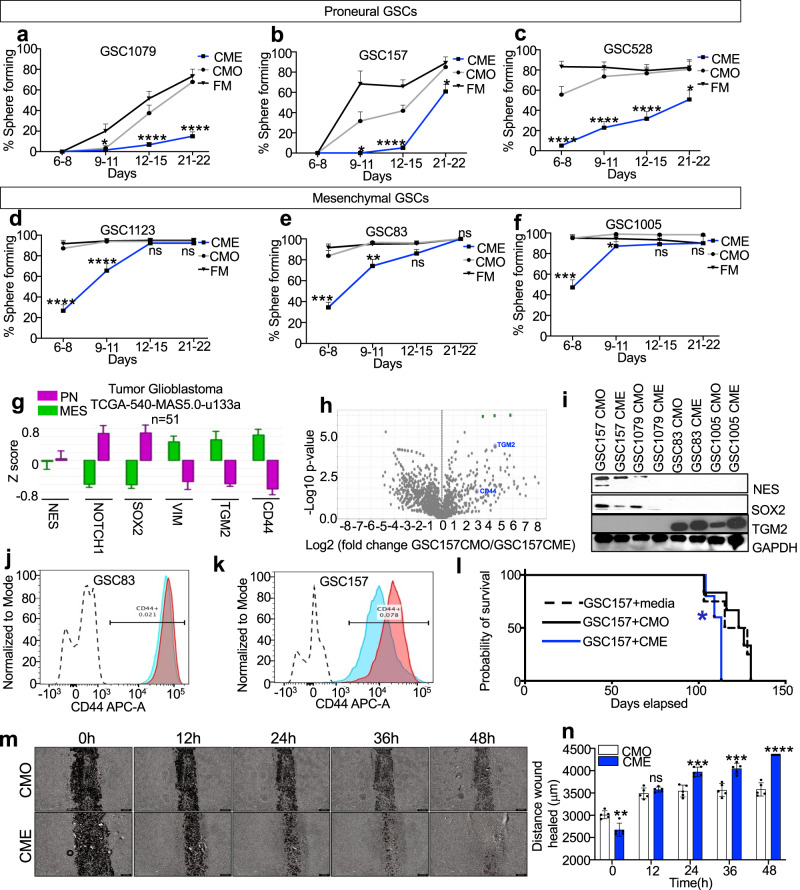
Mesenchymal reprogramming of proneural glioma cell stemness by endothelial conditioned media. Time-dependent changes in clonogenicity (ELDA) of GSCs in the presence of endothelial conditioned media (CME) or control media (CMO), compilation of limiting dilution assays across three proneural GSC lines: GSC1079 (**a**), GSC157 (**b**), GSC528 (**c**) and three mesenchymal GSCs lines: GSC1123 (**d**), GSC83 (**e**), GSC1005 (**f**); black lines represent cells treated with fresh media (FM), gray lines are indicative of cells treated with CMO, and blue lines represent cells treated with CME; *n* = 3 each group and each time point. **g** TCGA data mining of proneural versus mesenchymal genetic signatures across 51 patients diagnosed with GBM. Proneural hallmarks include, but are not limited to, NES, NOTCH1 and SOX2. Mesenchymal hallmarks include, but are not limited to, VIM, TGM2 and CD44. Green and purple represent mesenchymal (MES) and proneural (PN) genetic signatures, respectively. **h** Volcano plot showing enriched mesenchymal markers detected in the mass spectrometry (MS) of CME treated GSC157 cells relative to CMO treated GSC157 cells. **i** Expression of proneural hallmarks (NES and SOX2) and mesenchymal hallmark (TGM2) in proneural cells (GSC157, GSC1079) and mesenchymal cells (GSC83, GSC1005) after 7 day treatment with CMO, or CME. Flow cytometry of mesenchymal-GSC83 (**j**) and proneural-GSC157 (**k**) for CD44-APC. Dashed line, IgG control; blue curve, cells treated with CMO; red curve, cells treated with CME. **l** Kaplan–Meier symptom-free survival curve of tumor bearing mice injected with proneural-GSC157 cells pre-treated with either fresh media, CMO or CME; *n* = 5 mice per group; mice intracranially injected with cells pretreated with either fresh media (media) - dashed line, with CMO—black line, or with CME—blue line. **m** Wound healing/cell migration assay of proneural-GSC157 cells grown as monolayer and treated with CMO or CME throughout 48 h. **n** Quantification of the wound healing assay for CMO or CME treated GSC157 cells. GSC glioma stem cells, CMO own conditioned media, CME conditioned media derived from endothelial cells. Source data are provided as a Source Data file.

Clonal sphere formation is often regarded as a reflection of cancer cell “stemness”, which can be measured by extreme limiting dilution assay (ELDA). Cancer cell stemness is often enhanced by their interactions with endothelial cells^28^. However, in our hands, components of the endothelial cell secretome (CME) disrupted GSC sphere formation suggesting a possibility of a paradoxical (negative) impact on cellular clonogenicity. To explore this further, we conducted time-dependent ELDA screens of clonogenic proficiency in a series of proneural GSCs (GSC157, GSC1079, GSC528) and mesenchymal GSCs (GSC1123, GSC1005, GSC1123), which revealed considerable subtype-dependent differences. In line with their sphere disrupting effects, CME preparations markedly suppressed and delayed clonogenicity of proneural GSCs with a considerably less pronounced impact on their mesenchymal GSC counterparts (Fig. 3a–f; Supplementary Fig. 6). Apart from using own condition media as a control, we used conditioned media derived from non-endothelial cells such as HEK293 cells and an immortalized astrocytic cell line, NHA, neither of which perturbed sphere formation (Supplementary Figs. 5k and 9a). These results suggest that CME phenotypically alters the GSCs implicating a plausible modification of these cells at a molecular level.

GSC subtype assignment^1,30,31^ is largely guided by their resemblance of gene expression pattern described in TCGA and subsequent GBM classifications^7,32^ (Fig. 3g). In agreement with this system, proneural GSCs express elevated SOX2, NOTCH1 and NES while their mesenchymal counterparts are enriched for CD44 and TGM2^1^ (Supplementary Fig. 1g, h). Notably, CME treatment of proneural GSC attenuated the former markers while enhancing the latter, as evident from our mass spectrometry profiling (MS) (Fig. 3h), validated using western blots (Fig. 3i) and FACS (Fig. 3j, k).

Due to the altered phenotype, ELDA results and marker expression in the GSCs after CME treatment, we asked if this angiocrine impact would diminish the growth potential of these cells. However, when GSCs were incubated with CMO or CME for 3-7 days, the cells exhibited comparable levels of cellular growth and viability (Supplementary Fig. 7a–r), with modest differences between proneural and mesenchymal GSC subtypes. Moreover, despite reduced sphere formation, proneural GSCs exposed to CME were capable of initiating tumor growth in mice. Specifically, intracranial injection of mice with GSC157 cells pretreated with fresh media (FM), CMO or CME resulted in engraftment and development of the disease. Moreover, CME pre-treatment was associated with a shorter symptom-free survival of inoculated mice (Fig. 3l), suggesting that (in spite of ELDA results) endothelial secretome does not reduce the tumor initiating potential of proneural GSCs, but instead de-couples it from the proneural sphere forming ability.

Taken together, this pro-mesenchymal effect of the endothelial secretome reduced sphere formation, and induced some increase in cell proliferation, elongated cell shape, reduction of proneural GSC markers, expression of mesenchymal GSC markers and an apparent increase in tumor aggressiveness in vivo. In keeping with these findings, CME appears to induce mesenchymal transition of proneural GSCs which was also associated strongly with an increased cell motility (wound healing assay; Fig. 3m, n; Supplementary Fig. 7s, t) prompting our inquiry into the bioactive component present in the endothelial cell derived conditioned media.

### Endothelial cell derived extracellular vesicles mediate reprograming effects on proneural glioma stem cells

**Fig. 4:**
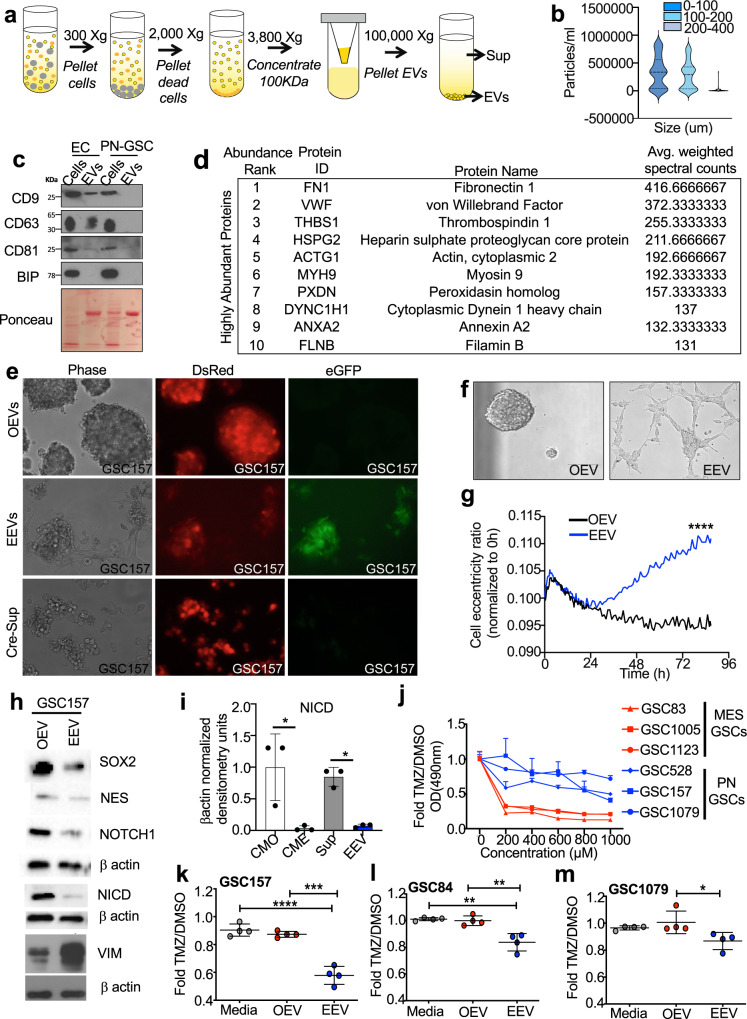
Extracellular vesicles recapitulate mesenchymal reprogramming effects of endothelial secretome against proneural glioma stem cells. **a** Schematic of extracellular vesicle (EV) isolation from conditioned media. **b** Nanoparticle tracking analysis (NTA/Nanosight) of EVs isolated from CME (HUVEC-CM). **c** Immunoblot for tetraspanins (CD9, CD63, CD81), and EV purity marker, BIP, indicates the absence of cytoplasmic contamination in EV preparations, according to MISEV2018^37^; Ponceau loading control. **d** Mass spectrometry analysis of the most abundant proteins enriched in HUVEC EVs (EEVs). **e** EV transfer assay: HUVEC cells were transduced with Cre-mCherry and proneural-GSC157 were transduced with dsRed/LoxP/eGFP lentiviral vectors. EVs from *Cre*-transduced HUVECs were purified and incubated with dual reporter proneural-GSC-157 for 4 days to observe a red to green fluorescence switch in recipient GSC157 cells. *Cre*-loxP experiment involving proneural-GSC157 cells treated with OEVs (top panel), *Cre*-EEVs (middle panel) and culture supernatant (Sup) from *Cre*-expressing cells (bottom panel). **f** Morphological differences observed in proneural-GSC157 cells treated with OEVs or HUVEC-EVs (EEVs) after 3 days in culture. **g** Cell eccentricity ratios measured by the Incucyte software for proneural-GSC157 cells treated with OEV (black line) versus cells treated with EEVs (blue line). **h** Expression of SOX2, NES, NOTCH1, NICD, and VIM in proneural-GSC157 cells treated with OEVs or EEVs. **i** Densitometry analysis of NICD expression (activated NOTCH1) in GSC157 cells treated with CMO, HUVEC-conditioned media (CME), CME derived EVs and Sup (*n* = 3). **j** Temozolomide (TMZ) dose-response curve for mesenchymal-GSCs and proneural-GSCs. MTS assay quantifying viability of proneural-GSCs after TMZ treatment in cultures pretreated with fresh media (media), OEV or EEV: GSC157 (**k**), GSC84 (**l**) and GSC1079 (**m**). NICD notch intracellular domain, CMO own conditioned media, Sup supernatant, EV extracellular vesicles, OEVs own EVs EEVs endothelial cell derived EVs, TMZ temozolomide, HUVEC human umbilical vein endothelial cells. Source data are provided as a Source Data file.

Since extracellular vesicles (EVs) play important roles in bidirectional tumor-stromal communication^33,34^ and are a part of endothelial secretome^35^, we reasoned that EVs may contribute to the mesenchymal transition driven by CME. To this end, CME was processed to separate endothelial cell extracellular vesicles (EEVs) and supernatant (Sup) using optimized ultracentrifugation protocol^36^ (Fig. 4a). The EEV fraction was further characterized using nanoparticle tracking (NTA) (Fig. 4b) along with immunodetection of tetraspanins (CD9, CD63, CD81) and purity marker (BIP)^37^ (Fig. 4c; absence of some of these markers in proneural-GSC EVs was described earlier^3^). Proteomic analysis of EEVs identified several highly enriched proteins including FN1 and HSPG2, which are involved in the EV uptake and induction of cell motility^36,38^ (Fig. 4d).

Since EVs often exert their biological effects through their uptake by recipient cells, EEVs might be expected to be internalized by their proneural GSC targets^36^. To ascertain whether this is the case, we adopted the previously developed *Cre*–*loxP* reporter system where EV mediated transfer of *Cre* recombinase triggers the expression of fluorescent proteins in recipient cells^39,40^. In this case, ECs were transduced with *Cre*-mCherry expressing lentivirus, while recipient GSC157 were transduced with a *DsRed/loxP/eGFP* dual Cre-sensitive reporter. Indeed, 4 days after exposure to Cre-EEVs the recipient GSC157 cells turned on eGFP expression (signifying *Cre-*EV uptake), which was not observed in the presence of control OEVs, or *Cre*-EC Supernatant (Fig. 4e). To further validate these findings, GSC157 cells were treated with EEVs labeled with fluorescent dye (CFSE) and imaged 24 h later using high resolution confocal microscopy and ImageStream cytometry (Supplementary Fig. 8a–c), in all cases revealing a robust uptake of EEVs.

To assess the possible angiocrine effects of the EEV uptake, sparse cultures of proneural GSC157 were treated with either EEVs or tumor own EVs (OEVs) and their responses were recorded using Incucyte in real time for up to 4 days (Supplementary movies 3 & 4; Supplementary Fig. 8d–o). While GSC157 cells incubated with OEVs remained stationary and grew as characteristic tight spheres with stable cell-cell contacts, the addition of EEVs disrupted this pattern in a manner indistinguishable from that of CME (Fig. 4f). Proneural GSCs grown in the presence of EEVs remained highly motile, adherent to substratum, exhibited notable protrusive activity and eventually formed a web like network (Fig. 4f). Incucyte recordings processed for cell eccentricity also supported the EEV-dependent stimulation of saltatory cell movements (round ➔ elongated ➔ round; Fig. 4g). Interestingly, treatment of proneural stem cells with control NHA-EVs did not disrupt the spheres or change phenotypes of these cells (Supplementary Fig. 9a). Since CME not only altered proneural GSCs phenotypically, but also molecularly (Fig. 3), we reasoned that EEVs impose a similar effect on these cells.

In order to assess the molecular impact of EEVs on their proneural GSC recipients, we profiled the protein signatures of proneural GSCs after EEV exposure. As expected, and similar to CME effects, signals for SOX2, NES and NOTCH1 were reduced in cells exposed to EEVs, but not to OEVs (Fig. 4h). We also observed a downregulation of NOTCH pathway signaling activity as reflected in a reduced levels of NOTCH intracellular domain (NICD) (Fig. 4h,i; Supplementary Fig. 9c). Interestingly, VIM and CD44 were found to be upregulated in proneural cells treated with EEVs (Fig. 4h, Supplementary Fig. 9b), not unlike following CME treatment (Fig. 3k). Immunofluorescence of proneural GSCs treated with EEVs also revealed morphological changes in combination with higher levels of mesenchymal proteins, such as aSMA and VIM (Supplementary Fig. 9d–g), and lowered levels of KRT31, thus providing additional evidence as to the ability of EEVs to impose a more mesenchymal fate upon the proneural GSC phenotype (Supplementary Fig. 9f, g). However, no obvious features of astrocytic or endothelial differentiation were apparent during EEV-induced mesenchymal transition of proneural GSCs suggesting a restricted nature of their reprogramming (Supplementary Fig. 9h–j).

Transitions between mesenchymal and proneural phenotypes is known to impact the sensitivity of cells to temozolomide (TMZ) chemotherapy, with mesenchymal GSCs being more sensitive relative to their proneural counterparts^1,4,15^ (Fig. 4j). Therefore, we asked whether the responses of proneural GSCs to TMZ would change in the presence of EEVs. Interestingly, we observed that while three different proneural GSC lines were, as expected, relatively refractory to this agent, their cytotoxic responses were exacerbated in the presence of EEVs relative to cultures in fresh media or OEVs (Fig. 4k–m). This is consistent with the reported drug responses of mesenchymal GSCs^4^ and suggestive of a role that endothelial vesicles may play in modulating the effects of anticancer agents in GBM.

The molecular control of mesenchymal traits imparted upon proneural GSCs in the presence of EEVs is of considerable interest. Since, during this process we observed a coordinated loss of NICD and SOX2 expression, the regulation of which could be epistatically linked^41^, we blocked NOTCH pathway in proneural GSC157 cells using γ-secretase inhibitor, DAPT (Supplementary Fig. 10a). This resulted in the expected downregulation of NICD and SOX2, but did not increase the VIM expression, suggesting that NOTCH inhibition is insufficient to recapitulate the full spectrum of mesenchymal changes induced by EEVs in proneural GSCs (Supplementary Fig. 10b). Indeed, such changes could be multifactorial given the molecular complexity of EEVs.

### Matrix metalloproteinases in endothelial extracellular vesicles alter the signaling programs of proneural glioma stem cells

**Fig. 5:**
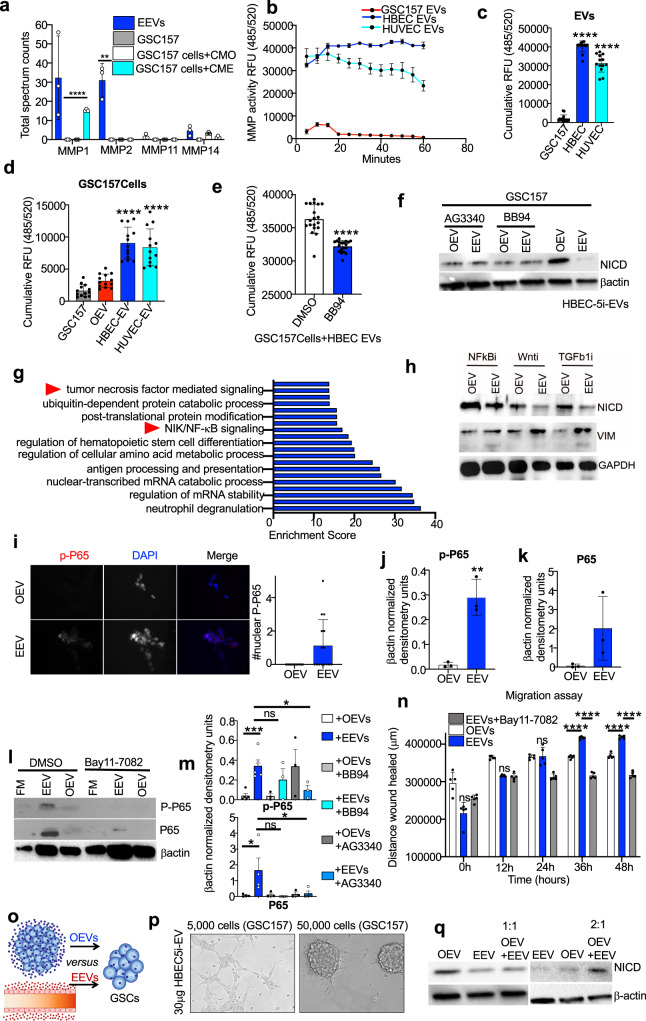
Extracellular vesicles impact multiple signaling elements of the glioma stem cell program. **a** Mass spectrometry quantification (*n* = 3) for the presence of MMPs in HUVEC-EVs (EEVs), proneural-GSC157 cells, GSC157 cells treated with CMO and GSC157 cells treated with HUVEC-CM (CME). Comparisons are made relative to untreated GSC157 cells (**b**, **c**). MMP activity of GSC157 EVs, HUVEC-EVs and HBEC5i EVs over 1 h (*n* = 3; **b**) and cumulatively (*n* = 3; **c**). **d** Cumulative MMP activity for GSC157 cells, GSC157 cells treated with their own EVs (OEVs), HBEC5i EVs and HUVEC-EVs over 1 h (*n* = 3). **e** Cumulative MMP activity of GSC157 cells treated with HBEC5i-EVs and either DMSO control or MMP inhibitor, BB94 (*n* = 3). **f** Expression of NICD following treatment of proneural-GSC157 cells with OEVs or EEVs in the absence or presence of MMP inhibitors, AG3340 and BB94. **g** DAVID analysis of the top pathways enriched in proneural-GSC157 cells treated with CME (HUVEC-conditioned media). **h** Expression of proneural-(NICD) and mesenchymal-(VIM) hallmarks after pharmacological inhibition of: NFκB pathway (Bay11-7082), Wnt pathway (LGK974) and TGFβ pathway (LY2157299). **i** Immunocytochemistry and quantification of phospho-P65 (p-P65) after treatment of GSC157 cells with EEVs (HUVEC-EVs). Densitometry quantifications of p-P65 (**j**) and total P65 (**k**) in GSC157 cells treated with HBEC5i-EVs (EEVs) relative to OEVs (*n* = 3). **l** Expression of activated (phospho) and total p65 in the presence of Bay11-7082 in GSC157s. **m** Expression of activated (phospho) and total p65 in the presence of AG3340 and BB94 in GSC157 cells exposed to EEVs. **n** Quantification of wound healing assay for OEV, EEV or EEVs+Bay 11-7082 treated proneural-GSC157 cells. **o** Schematic of the different donor EVs (OEVs and EEVs) competing for being taken up by proneural-GSC157. **p** Either 5,000 or 50,000 GSC157 cells exposed to a fixed amount of 30μg of HBEC5i-EVs (EEVs). **q** Expression of NICD in the presence of OEVs, HUVEC-EVs (EEVs), OEV:EEV (1:1) and OEV:EEV (2:1). NICD Notch intracellular domain, CMO own conditioned meida, CME endothelial conditioned media, EV extracellular vesicles, OEVs own EVs, EEVs endothelial cell derived EVs, MMP matrix metaloprotinase; HUVEC human umbilical vein endothelial cells, HBEC5i immortalized human brain brain endothelial cells. Source data are provided as a Source Data file.

We reasoned that EEVs may act at several levels on the sphere forming process and phenotype maintenance by proneural GSCs from forming multicellular spheres to signaling (Supplementary Fig. 10c). In this regard, matrix metalloproteinases (MMPs) are of interest since they are known products of endothelial cells and validated cargo of their EVs^42,43^. We hypothesized that MMPs may have a potential to disrupt sphere formation, and also they have a documented role in triggering differentiation of neural progenitor cells^44^, with the possible contribution to angiocrine EEV effects we observed. Indeed, we identified several MMPs in endothelial EVs (Fig. 5a; Supplementary Fig. 10c), but only MMP1 was upregulated in the proteome of GSC157 cells treated with CME (Fig. 5a). Moreover, direct measurements revealed high MMP activities in lysates of both HUVEC- and HBEC5i-EVs, but not in proneural GSC157 EVs (Fig. 5b, c). Similarly, untreated GSC157 cells themselves exhibited low MMP activity, but this activity rose dramatically following the exposure of these cells to HUVEC- or HBEC5i-EVs (Fig. 5a,d; Supplementary Fig. 10d). Next, we inhibited MMP activity in proneural GSC157 cells treated with EEVs using the BB94 compound^45^ (Fig. 5e, f; Supplementary Fig. 10e), which blocked downregulation of the NOTCH1 activity by EEVs. Taken together, our findings suggest that MMPs are transported as cargo of endothelial vesicles to proneural GSCs where they contribute to mesenchymal reprograming of these cells.

Since we previously observed that NOTCH signaling is not singularly responsible for mesenchymal reprogramming of proneural GSCs, we sought to explore other candidate stem cell pathways, including previously implicated NFκB^15^, Wnt^46^ and TGF-β^47^ cascades. Notably, some of these pathways were enriched in proteomes of proneural GSC157 cells treated with CME relative to CMO (Fig. 5g; Supplementary Fig. 10f). Pharmacological inhibition of these three pathways in EEV-treated GSC157 cultures (Fig. 5h) revealed that only NFκB inhibitor (Bay11-7082), not only blocked the downregulation of the NOTCH1 activity (loss of NICD), but also imparted upregulation of VIM. Interestingly, NFκB is known to be triggered following the uptake of EVs in other cancer cells^48^. In keeping with this notion, we observed an increased expression and phosphorylation of the P65 subunit of NFκB in GSC157 cells treated with EEVs (Fig. 5i–k; Supplementary Fig. 10g, h), and this effect was suppressed by Bay11-7082 treatment (Fig. 5l). Interestingly, inhibiting MMP activity reduced P65 levels in proneural GSCs treated with EEVs suggesting that NFκB is, in fact, downstream of MMP activity during transition of proneural GSCs to a mesenchymal-like state after the EEV uptake (Fig. 5m). Since mesenchymal transition would be expected to stimulate cell motility, we also performed migration (wound healing) assays, which revealed a markedly increased GSC movement in the presence of EEVs, which was suppressed by addition of Bay11-7082 (Fig. 5n; Supplementary Fig. 10i). Collectively, these results suggest that the complex effects of EEVs converge upon the NFκB pathway in proneural GSCs (after EEV uptake) resulting in their multifaceted mesenchymal transition.

EV uptake often depends on their interaction with heparin sulfate proteoglycan (HSPG) on recipient cell surface^49–51^. Therefore, we asked whether blocking this interaction with heparin would be sufficient to impede the onset of mesenchymal reprograming of proneural GSCs in the presence of EEVs. Indeed, heparin treatment significantly inhibited EEV internalization by GSC157 cells and prevented both the downregulation of NOTCH1 activity and increase in VIM expression (Supplementary Fig. 11 a, b). In contrast, stripping surface proteins from EEVs by exposure to 1 M KCl^50,51^ did not dimmish their effects further suggesting that EEV internalisation (rather than surface-surface interaction) is an important part of their effect on proneural GSCs (Supplementary Fig. 11b). Collectively, our findings suggest that angiocrine EEV uptake mediates the increase in MMP activity in proneural glioma stem cells, which leads to activation of NFκB signaling pathway thereby inducing increased cell migration and the onset of other mesenchymal-like traits.

### Endothelial extracellular vesicles compete with tumor vesicles for influence on cancer cells

Growth pattern of GBM cells (and GSCs) may lead to their homing to tumor core, edge, and distant brain parenchyma, including perivascular regions, each with considerable consequences to tumor-tumor and tumor-vascular interactions^6,18^. It is therefore plausible that the homotypic cell-cell interactions of GSC157 cells, or abundance of GSC EVs (OEVs) facilitate the juxtacrine NOTCH signaling and maintenance of the proneural phenotype (with high hNICD levels). This process could be disrupted by excess of competing EEVs when cancer cells infiltrate the surrounding brain (Fig. 5o), especially since proneural GSCs somewhat preferentially take up EEVs relative to OEVs (Supplementary Fig. 11a). To test this interplay between homotypic and heterotypic EV-cell interactions, fixed amount (30μg) of EEVs was added to either low (5000) or excessive (50,000) numbers of GSC157 cells. After three days in culture, in the former condition, GSC157 underwent the aforementioned mesenchymal change and adhered onto the culture dish as cellular networks. In contrast, excessive numbers of cells remained virtually unaffected by fixed EEV numbers, and the cells grew as spheres with minimal adhesion and network formation (Fig. 5p). To understand whether this could be due to sustained cell-cell contact or accumulating OEVs, the excess of OEVs was mixed with EEVs and added to GSC157 cultures at a ratio of 2:1. In this setting, the EEV-dependent NICD loss was completely prevented, but not when the EEV:OEV ratio was set at 1:1 (Fig. 5q). This suggests that cancer cell responses may vary according to competitive influences of various EV populations present in their surroundings.

### Angiocrine stimulation promotes brain invasion by glioma stem cells

**Fig. 6:**
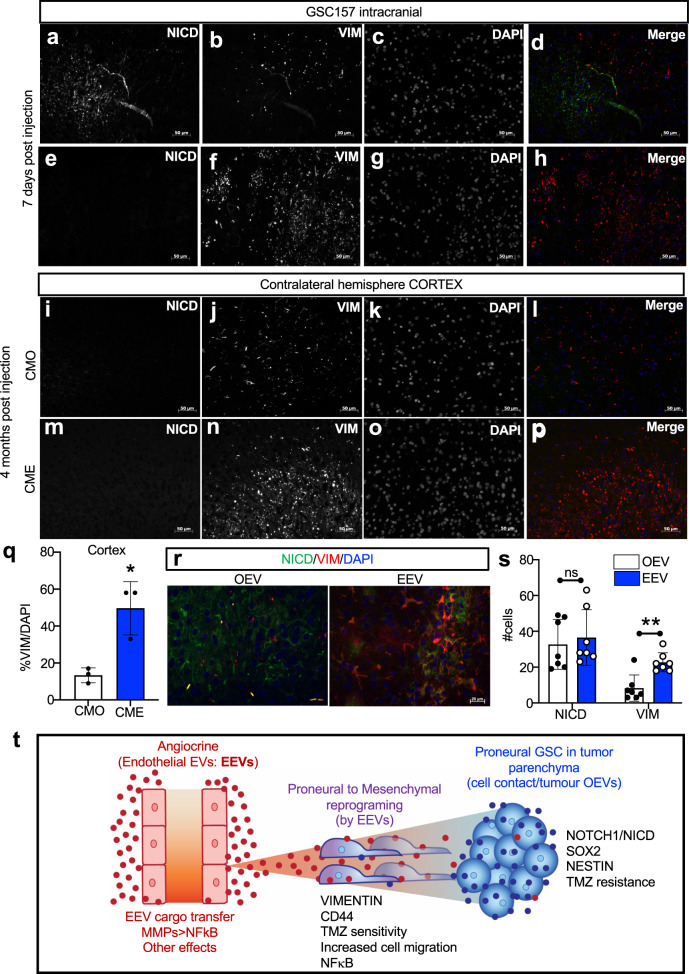
Enhancement of glioma stem cell invasion of the brain following exposure to endothelial cell secretome. **a**–**h** Proneural-GSCs were tracked in the brain of mice 7 days after intracranial injection; tissues were stained for human antigens: hNICD and hVIM. Image is taken at the tumor site. **i**–**p** Expression of hNICD and hVIM in the cortex of the contralateral hemisphere in mice intracranially injected with proneural-GSC157 cells pretreated with CMO **i**–**l** and CME **m**–**p** after 4 months post inoculation. **q** Quantification of %VIM/DAPI of proneural-GSC157 cells pretreated with CMO or CME in the cortex (*n* = 5). **r** Expression of hNICD and hVIM at the tumor site in the brains of mice 7 days after intracranial injections of proneural-GSC157 cells pretreated with OEVs or EEVs. **s** Quantification of the number of cells positive for hNICD and hVIM in the xenografts of proneural-GSC157 cells pretreated with OEVs or EEVs. **t** Model summarising the influence of endothelial EVs on mesenchymal reprogramming of proneural GSCs. EEVs carrying MMPs and possibly other bioactive cargo initiate reprograming events leading to downregulation of proneural hallmarks and upregulation of mesenchymal regulators, including NFκB. The model proposes that EEVs compete with homotypic interactions (OEVs and cell-cell contact) for influence over the phenotype of glioma stem cells upon which they impose a mesenchymal-like program. CMO own conditioned media, CME conditioned media derived from endothelial cells, OEVs own extracellular vesicles, EEVs endothelial cell derived extracellular vesicles.

Our results predict that in the presence of endothelial cells, solitary proneural GSCs often observed in the brain, with less exposure to their own EVs may become prone to mesenchymal transition and enhanced invasiveness in vivo. To examine this possibility, proneural GSCs were implanted orthotopically into the striata of NSG mice and their fate and phenotypes were followed over time. Interestingly, as early as 7 days post implantation, the fates of GSCs began to diverge (Fig. 6a–h) with cells in the post-inoculation mass (with close homotypic cell-cell contacts) being strongly positive for human NICD (hNICD) and negative for human VIM (hVIM), similar to the GSC spheres or in the presence of abundant OEVs. In contrast, the solitary GSCs scattered around the injection site assumed an antithetical, hNICD-negative and hVIM-positive, mesenchymal-like phenotype, with virtually no overlap between these populations (Fig. 6a–h). To assess whether the latter cells may represent a migratory/invasive phenotype inducible by the endothelial secretome, proneural GSCs157 were pre-treated with conditioned media - CME or CMO (containing the respective EVs) prior to intracranial inoculation. Once again, the hNICD-positive and hVIM-positive populations readily emerged, with the latter cells readily penetrating distant regions of the brain. These remote brain regions included the cortex and corpus callosum with greater preponderance of the GSC invasion (hVIM^+^) in the case of inoculates pre-treated with CME, relative to CMO (Fig. 6i–q, Supplementary Fig. 12). We also observed a higher number of hVIM expressing cells in GSC157 cells in vivo if they were pretreated with EEVs in comparison to OEV pretreatment (Fig. 6r, s). Thus, the availability of homotypic and heterotypic EV populations and their respective uptake may promote either proneural or mesenchymal fate of glioma stem cells.

## Discussion

Taken together, this work documents that endothelial secretome, including EVs, alters the nature of GSC stemness in a manner dependent on the molecular subtype of these crucial tumor initiating cells. Our observations emphasize the role of the NOTCH pathway in maintaining proneural tumor cell features within the tumor mass, which likely includes both perivascular niches and the cellular networks detached form the vasculature, as recently described^18^. However, perivascular interactions of proneural GSC in the distant brain parenchyma may occur largely in the context of downregulated NOTCH activity and in the presence of mesenchymal traits, such as high expression of VIM. Indeed, endothelial cells may contribute to such mesenchymal reprogramming of proneural GSCs, a switch which changes their morphology, phenotype, and drug responsiveness (Fig. 6t), while promoting their migratory properties. As these cells scatter away from the tumor mass largely along vascular tracks, they may become increasingly susceptible to heterologous stromal and angiocrine influences (Supplementary Fig. 12z'). This interaction is mediated, at least in part, by angiocrine EVs, which could, to some extent, contribute to the infiltrative growth pattern observed in GBM, a feature which compromises the efficacy of local therapies (surgery, radiation) (Supplementary Fig. 12), but may also change the responsiveness to chemotherapy with temozolomide. It could be postulated that a tailored modulation of endothelial cell vesiculation processes, EV uptake, or related signaling responses could lead to a better control of the disease aggressiveness, at least in some subsets of GBM.

## Methods

### Cell culture

Patient derived GSC lines, namely, (GSC) 83, 1123, 1005, 528, 157, 84 and 1079 were isolated from GBM surgical samples, obtained in the laboratory of Dr. Ichiro Nakano. All the cell lines were grown in serum free media in suspension. The GSC culture media (fresh media, FM) comprised of DMEM-F12 basal media (GIBCO#11320033), EGF (GIBCO#PHG0311L), FGF (GIBCO#PHG0261), Heparin 0.2% (STEMCELL#07980), B27 (GIBCO#17504044), Glutamax (GIBCO#35050061) and 1% Penicillin-streptomycin (GIBCO#15070063). The gene and protein expression studies are periodically performed in house^4^ to confirm the identity of cell lines as published^52^, including RNA seq, staining for pluripotency markers and differentiation capacity. Human umbilical vein endothelial cells (HUVECs) and immortalized human brain endothelial cell line (HBEC-5i) were purchased from American Type Culture Collection (ATCC; Manassas, VA; #PCS-100-010 and #CRL-3245 respectively). Human primary brain microvascular endothelial cells (BMECs) were purchased from Cell Biologics# H-6023. HUVECs and BMECs were cultured in bullet kit media and supplements (Lonza#CC-3162). HBEC-5i were cultured in DMEM-F12 media supplemented with 10% FBS, 1% P/S and 40 μg/mL endothelial growth supplement (ECGS) (Sigma E2759). All the endothelial cells were grown as monolayers on 0.1% Gelatin (Sigma#G9391). HEK-293 (ATCC, #CRL-1573) and NHA (obtained from late Dr. Guha University of Toronto, and validated in house^53^) were cultured in DMEM media supplemented with 10% FBS and 1% Penicillin-streptomycin. All the cell lines purchased from ATCC or Cell Biologics were tested and validated by the respective company. Depleted media used to condition the cells and isolate their secretomes comprised DMEM-F12 and 0.125% EV-depleted FBS. GSCs, HUVECs, HBEC-5i and BMECs were cultured in depleted media for 3 days in vitro to obtain GSC specific conditioned media (own condition media, OCM) and HUVEC/HBEC5i/BMEC specific conditioned media (endothelial conditioned media, ECM). All the conditioned media (CM) were subjected to a 2000 X g centrifugation for 20 min to sediment cell debris prior to use. For all CM experiments, GSCs were cultured in either OCM or ECM at a 1:1 ratio with FM. Most of the CM experiments were 7 days long, unless mentioned otherwise. All cells were cultured in a 37 ^o^C incubator with 5% CO_2_. For co-culture assay, monolayered HUVECs were labeled with carboxyfluorescein succinimidyl ester (CFSE) (ThermoFisher#C34554) and GSCs labeled with PKH26 dye (Sigma# PKH26GL-1KT) were added in a 1:1 ratio and cells were cultured for 7 days.

### EV depleted FBS

FBS was centrifuged at 100,000 × *g* for 19 h to pellet EVs. Supernatant FBS was filtered through a 0.2 µm filter. This FBS was considered EV depleted. It was used to prepare starvation media for CM experiments.

### Inhibitors

The following inhibitors were used, as indicated: MMP inhibitors - BB94 (Selleckchem cat#S7155; 20 nM), AG3340 (Sigma cat#PZ0198; 10μM), NFkB inhibitor—Bay11-7082 (Selleckchem cat#S2913; 0.5 μM), NOTCH inhibitor—DAPT (Sigma cat#D5942; concentration as per supplementary Fig. 10), Wnt inhibitor—LGK-974 (Selleckchem cat#S7143; 2 μM), TGFb pathway inhibitor—LY2157299 (Selleckchem cat#S2230; 10 μM), Proteoglycan EV receptor inhibitor—Heparin (Stemcell Technologies cat#7980, 10 μg/ml).

### Real time imaging using Incucyte

GSCs were plated in 24 well dishes in either CMO or CME and placed in an Incucyte S3 live analysis system (EssenBio). Images were obtained at 10 X every 30 min for 7 days. Cell sharpness (cell adherence) and eccentricity (change in cellular morphology) were analyzed using the standard Incucyte analysis software. Images were acquired using bright field, red and green fluorescence.

### xCelligence analysis

The xCelligence RTCA MP instrument (ACEA Biosciences) was used to measure cell impedance (cell adherence). The E-plate VIEW 16 (ACEA Biosciences# 6324738001) was used to plate cells at a clonal density in OCM or ECM cultured for 7 days.

### Extreme limiting dilution assay (ELDA)

Cells were seeded at different densities (1–40 cells/well) in 96 well plates. Up to 12 wells for 1 cell and 6 wells per 5-, 10-, 15-, 20-, 25-, 30-, 35-, and 40-cells were seeded using FACS. Cells were cultured in 100 μL of either CME (1:1, FM:CME), CMO (1:1, FM:CMO), or FM for 1–6 weeks as indicated. Cells were fed every 15 days with 100 μL of respective CM/FM media. At each timepoint, the number of wells with at least one tumor sphere (clusters/spheres comprising of >20 cells) were counted. The frequency of cluster-initiating cells was calculated by an ELDA online program at http://bioinf.wehi.edu.au/software/elda/. Endpoint was reached once all wells contained a tumor sphere, or if no additional tumor spheres formed after 3-7 additional days of incubation post endpoint.

### Cell growth/survival assay (MTS assay)

500 cells/well of a given cell-line were seeded in 96 well plates with 100 μL of either CME (1:1, FM:CME) or CMO (1:1, FM:CMO) media to afford 5 technical replicates (*n* = 5). Cells were left to incubate for 3, 5, or 7 days as indicated. MTS reagent corresponding to 1/10th the total volume present in a given well was used to measure cell growth/survival in presence/absence of endothelial-cell (HUVEC) conditioned media. After 2 h of treatment, the absorbance at 490 nm was read using a plate-reader. The readings were normalized to the MTS readings obtained using CMO alone and CME alone without the presence of cells. MTS for temozolomide (TMZ, S-1237, Selleckchem) was done after 24 h incubation with TMZ.

### Intracranial injections, Lycopersicon lectin Injection and Vibratome Sectioning

Orthotopic injections were carried out by injecting 2 μl of 25,000 GSCs stereotactically into the brains of 3 month old NOD.Cg-PrkdcSCID Il2rgtm1Wjl/SzJ (NSG) mice (Charles River Labs). For staining blood vessels, *Lycopersicon lectin* (DL-1178, vector Laboratories) was injected i.v. 30 m prior to euthanizing the mouse. Brains from mice were harvested and put in cold PBS. The brains were sectioned at 200 μm using a vibratome (Leica VT 1200 s). The tissues were placed in a µ-Dish 35 mm, high Glass Bottom dish (81158, ibidi) and taken for high resolution confocal microscopy (Zeiss LSM780 laser scanning confocal microscope). An end point of a mouse with intracranial injection was established based on the first sign of neurological symptoms such as circling or dehydration. No decline in well being was allowed. All mice were maintained at MUHC RI and McGill University animal care facility, monitored daily, under 12 h of light/12 h of dark cycle. All procedures comprising animals were conducted in accordance with the guidelines of the Canadian Council of Animal Care and the Animal Utilization Protocols, approved by the Institutional Animal Care Committee at the Research Institute of the McGill University Health Center and McGill University.

### Sprouting assay with GSC co-culture

Aortic ring sprouts were prepared as described previously^54^. Briefly, aortas of 2 month old C57bl/6 (Charles River Labs) mice were collected and ~1 mm rings were cut and placed in the BME matrix (3533-005-02, Cultrex RGF BME, Type 2, R&D Systems) in a 12-well dish. After the ring solidified in the BME, aortic ring media containing DMEM/F12 (11320-033, GIBCO), 1X Penstrep (LS15140148, GIBCO), 2% FBS (080-150, Wisent) and ECGS (E2759, Sigma) were added to cultures. After 3d, when the endothelial cell sprouts developed, GFP labeled GSCs were incorporated in BME and placed at the edge of the well containing aortic rings. Images were taken 3 days after co-culture initiation.

### Scratch cell motility assay

GSCs were grown as monolayers in 12-well dishes coated with poly-l-ornithine (P4957, Sigma) and laminin (L2020, Sigma). A scratch was generated by running a P1000 sterile pipette tip across the confluent GSC culture. Respective media was added in each well and images were recorded over time using Incucyte S3 live analysis system (EssenBio).

### EV purification

EVs were purified as previously described^3^. Briefly, the respective conditioned media were cleared of cells at 300 × *g* for 5 min, and dead cells/debris was further eliminated by centrifugation at 2000 × *g* for 20 min, after which the supernatant was concentrated by spinning at 3500 × *g* for 20 min using Amicon Ultra-15 Centrifugal Filter Units −100 KDa- (UFC905008, Millipore) to a final volume of 1 ml. The 1 ml Concentrate was subjected to ultracentrifugation at 110,000 × *g* for 1 h using a tabletop ultracentrifuge (Beckman TLA100.2 rotor). The pelleted EVs were washed with sterile PBS (311-425-CL, Wisent) at 110,000 × *g* for 1 h in the ultracentrifuge suspended in sterile PBS or RIPA buffer and analysed.

### Nanoparticle tracking analysis (NTA)

Particle number and size were investigated using NS500 nanoparticle tracking analysis system (NTA-Nanosight, Amesbury, UK). 1:1000 aliquots were loaded into the chamber. Three recordings of 30 s each were obtained under automatic detection and processing settings at 37 °C with camera level at 15, detection threshold of 5 and blur size set by auto by NTA software (version 3.0).

### Plasmid constructs, expression vectors and cell transduction

pLV-CMV-LoxP-DsRed-LoxP-eGFP (plasmid 65726, Addgene) lentiviral vector was transduced into the PN-GSC157 cells. pLM-CMV-R-Cre (plasmid 27546, Addgene) lentiviral vector was transduced into HUVECs. Briefly, cells were plated at 10,000 cells per well in a 24 well dish. Based on titration, 10μl of the prepared virus was added to the cells and subjected to spinoculation at 800 × *g* for 1 h 30 m. After overnight incubation, respective media was changed. Three days following spinoculation, cells were washed three times in sterile PBS and cells exhibiting red fluorescence were sorted by FACS in the case of both, GSCs and HUVECs.

### Western blotting and proteomic analysis

Total cell proteins and EV proteins were isolated using RIPA buffer (10 mM tris at pH8.0, 1 mM EDTA, 1% triton X100, 0.1% sodium deoxycholate, 0.1% SDS, 140 mM NaCl) containing protease inhibitor (Roche, Mississauga, ON, Canada). After incubation on ice for 30 m, the lysates were centrifuged at 15,000 x g for 5 m at 4^o^C. Protein concentrations were assessed using the Pierce Micro BCA^TM^ Protein Assay (Thermo Scientific, Rockford, IL, USA). Using 10% sodium dodecyl sulfate–polyacrylamide gel electrophoresis (SDS-PAGE), lysates were resolved. Transfer of the resolved proteins onto polyvinylidene difluoride membranes (PVDF; Biorad, Mississauga, ON, Canada) was performed at 30 V overnight. Membranes were blocked for 1 h in 5% milk or 5% BSA and probed with primary antibodies and respective horseradish peroxidise (HRP)-conjugated secondary anti-mouse (170–6516, BioRad, 1:500), anti-rabbit (7074 S, Cell signaling, 1:500) or anti-goat (sc-2020, Santa Cruz, 1:500) antibodies. Chemiluminescence (GE Healthcare) was visualized using ChemiDoc MP system (Biorad). Primary antibodies used included: rabbit anti-CD63 (ab134045, Abcam or 556019, BD, 1:1000), rabbit anti-CD9 (ab92726, Abcam, 1:500), rabbit anti-CD81 (ab155760 Abcam, 1:100), rabbit anti-BIP (3183 S, Cell Signaling, 1:1000), rabbit anti-NOTCH1 (4380, cell signaling, 1:1000), rabbit anti-SOX2 (ab93689, Abcam, 1:100), rabbit anti-NES (ab105389, Abcam, 1:100), rabbit anti-TGM2 (3557 S, Cell Signaling, 1:1000), rabbit anti-NICD (07-1231, Millipore, 1:500), goat anti-VIM (AF2105-SP, R&D, 1:500), rabbit anti-P65 (8242 T, cell signaling, 1:1000), rabbit anti-pP65 (3033 S, cell signaling, 1:1000) and mouse anti-βactin (A1978, Sigma, 1:1000).

### Immunostaining

All brain tissues were preserved in 4% PFA after dissections. Tissues were passed through the tissue processor (Leica TP 1050) and embedded in paraffin blocks from which 5 μm thick sections were cut using a microtome (American Optical). Tissues were de-waxed and re-hydrated in a series of 5 min steps involving Xylene and 95%- >50% ethanol. Tissues were further processed by heating at 95^o^C for 15 min in unmasking solution (H3300, Vector Labs) for antigen retrieval. Primary antibodies: rabbit anti-CD31 (SAB5600061, Sigma, 1:1000) and mouse anti-NES (MAB1259, R&D, 1:100) were incubated overnight at 4^o^C. The tissues were washed for 5 min three times in PBS. The respective fluorescent secondary antibodies, purchased from Invitrogen, were incubated with tissues for 1 h at 37 ^o^C. Secondary antibodies: goat anti-rabbit IgG Alexa Fluor 488 (A-11034 Invitrogen, 1:500) and donkey anti-goat IgG Alexa Fluor Plus 594 (A32758 Invitrogen, 1:500). Tissues were washed for 10 min three times, then the slides were cover slipped using DAPI mounting reagent (VECTH1500, Vector Labs).

### Flow cytometry and ImageStream

GSC157 cultures were treated with CMO, CME, OEVs and EEVs for 7 days, then stained for CD44 (338805, Bio Legend) and IgG control. For the CFSE experiment, purified EVs were stained with CFSE (C34554, ThermoFisher) prior to incubation with cells. Cells were treated with CFSE labeled EVs for 24 h followed by FACS analysis. The cells were subjected to flow cytometry (BD LSR Fortessa) and data was analysed using the FlowJo software 10.7.1. For ImageStream, GSC157 cells were incubated with OEVs pre-stained with PKH26 (MINI67, Sigma) and EEVs pre-stained with DiD (V22887, Invitrogen). 24 h later, NucBlue (R37605, Invitrogen) was added and GSCs were subjected to Image Stream analysis (Amnis ImageStream Mark II).

### Mass spectrometry

Proteomics was done as previously described^3^. Briefly, data was collected using the Thermo Orbitrap Fusion mass spectrometer operating at 120,000 resolution (FWHM in MS1) with HCD sequencing (15,000 resolution). Data was analyzed using Prism 9. The raw data were converted into *.mgf format (Mascot generic format) for searching using the Mascot 2.6.2 search engine (Matrix Science) against Human Uniprot sequences (2020). The database search results were loaded onto Scaffold Q + Scaffold_4.9.0 (Proteome Sciences) for statistical treatment and data visualization. Analysis for biological pathways found in highly enriched proteins was performed using DAVID: https://david.ncifcrf.gov platform.

### MMP activity assay

Protein lysates (10-15 μg) were processed using the MMP analysis kit (ab112146, Abcam) as per the manufacturer’s instructions.

### Inhibition of EV internalization and stripping outer proteins of EEVs

EEVs were prevented from internalization by exposing the proneural-GSC157 cells to heparin (10 μg/ml) for 1 h prior to EEV treatment. EEVs were stripped off the outer proteins by treating 1.5 ml of EEVs with 1.5 ml of 2 M KCl solution for 30 min at 4 ^o^C with agitation. Preparations were then subjected to ultracentrifugation, 110,000 × *g* for 1 h to pellet the treated EEVs. GSC157 was then incubated with the KCl treated EEVs and analysed as indicated.

### Statistical analysis

Standard unbiased Student’s *t* test analysis was used to compare two groups while one way ANOVA was used for comparison of more than two groups of data sets using GraphPad Prism v9. XCelligence data are plotted as average and standard deviation of well replicates. Every experiment was independently repeated > /= 3 times (biological repeats) and each experiment had > /= 5 technical repeats. The results were considered not significant (NS) when *P* > 0.05, or significant when *P* < 0.05 (*); *P* < 0.01 (**); *P* < 0.001 (***) and *P* < 0.0001 (****). All graphs were plotted to show the standard deviation of replicates.

### Reporting summary

Further information on research design is available in the Nature Research Reporting Summary linked to this article.

## Supplementary information

Supplementary Information

Description of Additional Supplementary Files

Supplementary Movie 1

Supplementary Movie 2

Supplementary Movie 3

Supplementary Movie 4

Reporting Summary

## Data Availability

The mass spectrometry proteomics data have been deposited to the ProteomeXchange Consortium via the PRIDE partner repository with the dataset identifier PXD034819. All other raw data are available upon request. Source data are provided with this paper.

## Source data

Source Data
