# Supplementary material for: Angiocrine extracellular vesicles impose mesenchymal reprogramming upon proneural glioma stem cells
Source: Nat Commun. 2022 Sep 19;13:5494. doi: 10.1038/s41467-022-33235-7 (PMC9485157; doi:10.1038/s41467-022-33235-7)
Supplement: Supplementary file 1 — Supplementary Information [file 41467_2022_33235_MOESM1_ESM.pdf]

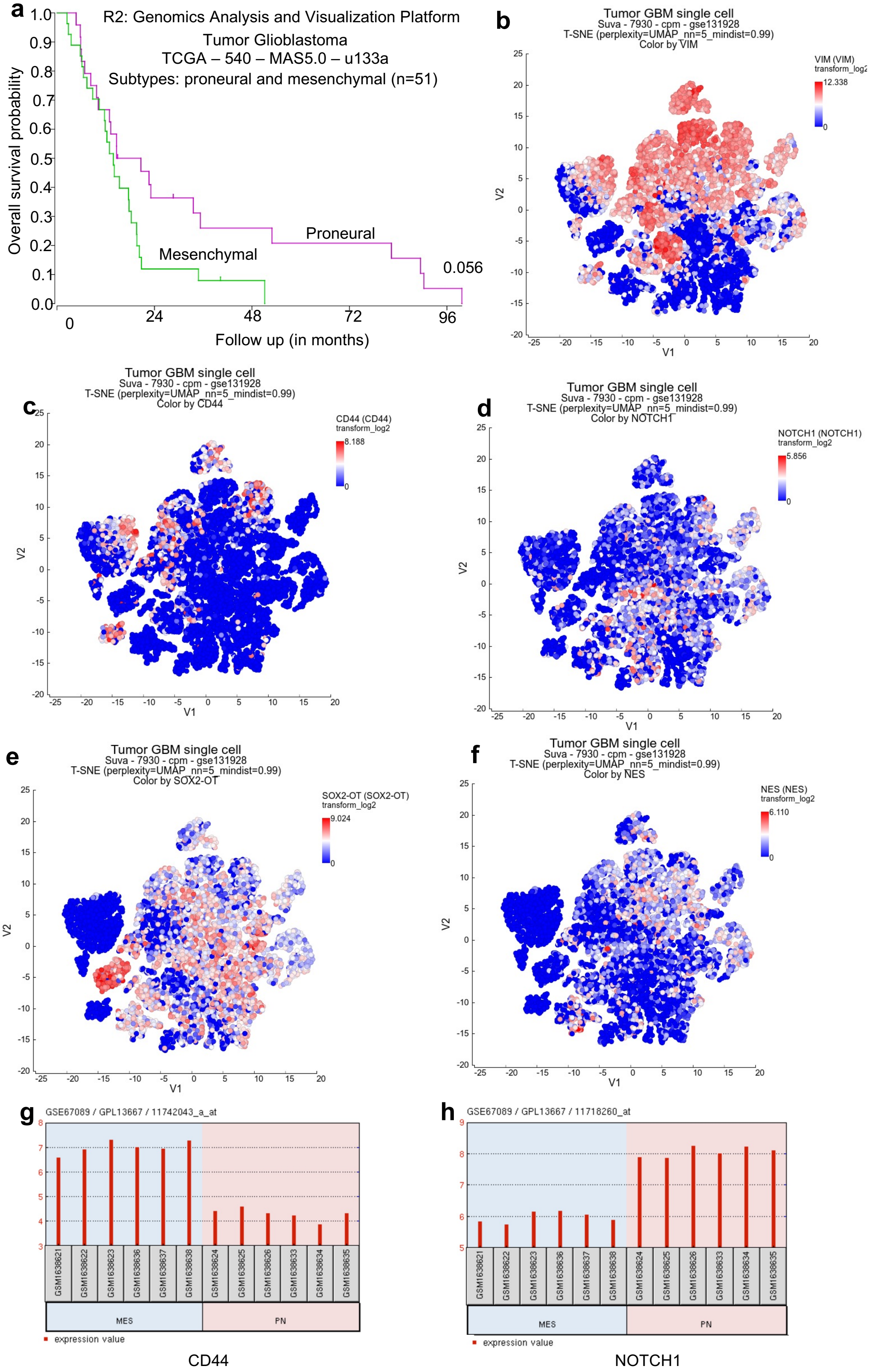
a
R2: Genomics Analysis and Visualization Platform
Tumor Glioblastoma
TCGA – 540 – MAS5.0 – u133a
Subtypes: proneural and mesenchymal (n=51)
Overall survival probability
Proneural
Mesenchymal
0.056
Follow up (in months)
b
Tumor GBM single cell
Suva - 7930 - cpm - gse131928
T-SNE (perplexity=UMAP_nn=5_mindist=0.99)
Color by VIM
VIM (VIM)
12.338
c
Tumor GBM single cell
Suva - 7930 - cpm - gse131928
T-SNE (perplexity=UMAP_nn=5_mindist=0.99)
Color by CD44
CD44 (CD44)
transform_log2
8.188
d
Tumor GBM single cell
Suva - 7930 - cpm - gse131928
T-SNE (perplexity=UMAP_nn=5_mindist=0.99)
Color by NOTCH1
NOTCH1 (NOTCH1)
transform_log2
5.856
e
Tumor GBM single cell
Suva - 7930 - cpm - gse131928
T-SNE (perplexity=UMAP_nn=5_mindist=0.99)
Color by SOX2-OT
SOX2-OT (SOX2-OT)
transform_log2
9.024
f
Tumor GBM single cell
Suva - 7930 - cpm - gse131928
T-SNE (perplexity=UMAP_nn=5_mindist=0.99)
Color by NES
NES (NES)
transform_log2
6.110
V1
V2
g
GSE67089 / GPL13667 / 11742043_a_at
MES
PN
expression value
CD44
h
GSE67089 / GPL13667 / 11718260_at
MES
PN
expression value
NOTCH1

Supplementary figure 1: *Human glioblastoma (GBM) and glioma stem cells (GSCs) exhibit distinct features*. (a) Kaplan Meier survival curve of 51 patients classified into proneural (pink) and mesenchymal (green) subtypes. (b-f) T-SNE plots of the sc-RNA dataset (from[8]) exemplifying a distinct cell population expressing mesenchymal markers VIM (b) and CD44 (c), and proneural markers, such as NOTCH1 (d), SOX2 (e) and NESTIN (f). (g,h) microarray dataset[1] of GSCs showing high expression of CD44 in mesenchymal glioma stem cells (g) and high expression of NOTCH1 in proneural glioma stem cells (h).

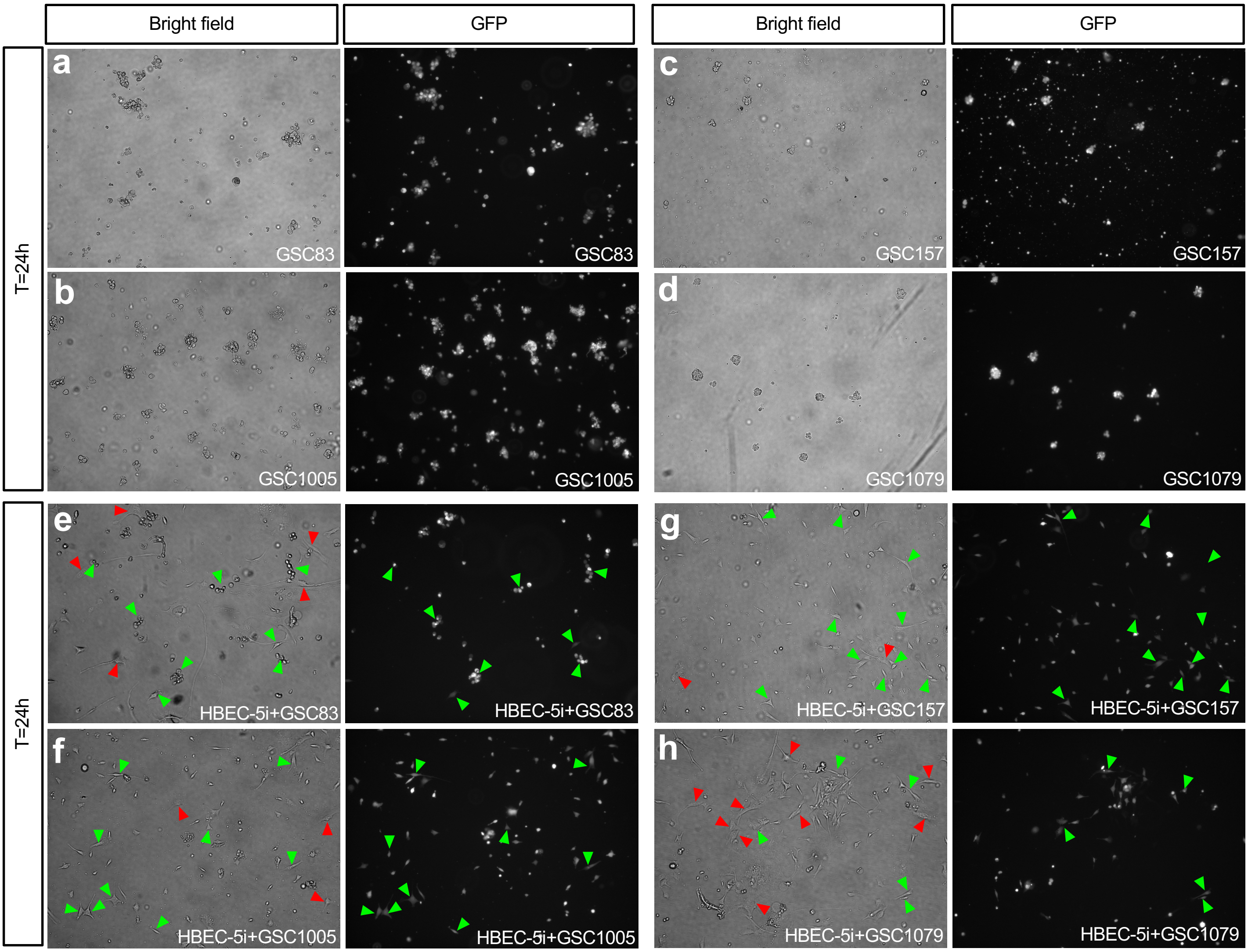

Supplementary figure 2: *Endothelial cell secretome causes reduction in GSC cluster formation*. (a,b) Three-dimensional growth of mesenchymal GSCs in serum-free media. Mesenchymal-GSC83 (a) and mesenchymal-GSC1005 cells (b) were imaged 24 hours after being cultured alone. (c,d) Proneural-GSC157 (c) and proneural-GSC1079 cells (d) 24 hours after being cultured alone. (e,f) Mesenchymal-GSC83 (e) and mesenchymal-GSS1005 cells (f) 24 hours after being co-cultured with HBEC-5i brain endothelial cells. (g,h) Proneural-GSC157 (g) and proneural-GSC1079 cells (h) 24 hours after being co-cultured with HBEC-5i brain endothelial cells. Green arrowheads point GSCs and red arrowheads point HBEC5i cells; HBEC5i, immortalized human brain endothelial cells.

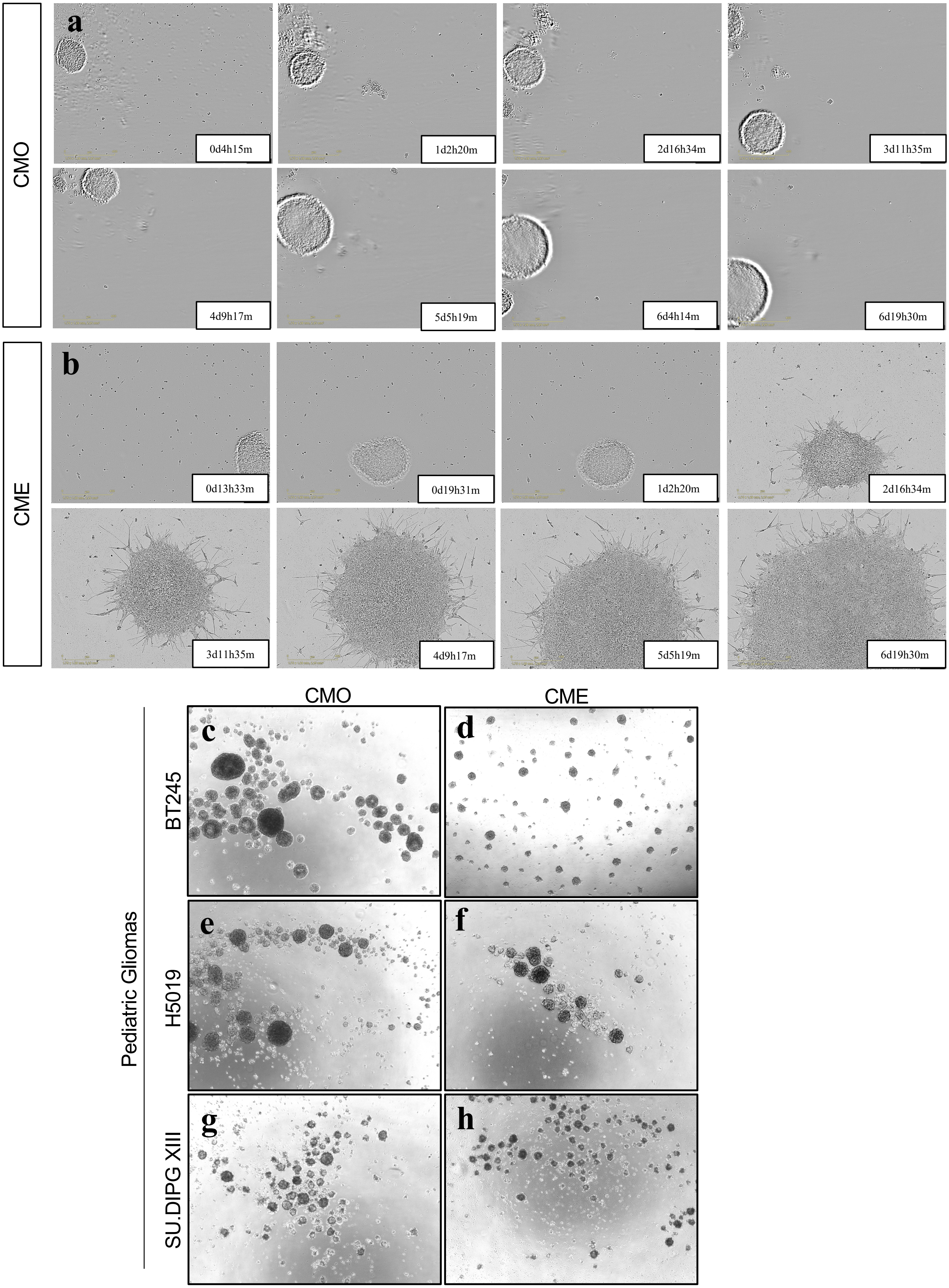
CMO
a
0d4h15m
1d2h20m
2d16h34m
3d11h35m
4d9h17m
5d5h19m
6d4h14m
6d19h30m
CME
b
0d13h33m
0d19h31m
1d2h20m
2d16h34m
3d11h35m
4d9h17m
5d5h19m
6d19h30m
CMO
CME
Pediatric Gliomas
BT245
c
d
H5019
e
f
SU.DIPG XIII
g
h

Supplementary figure 3: *Endothelial cell secretome triggers subtype-specific 'desphering' of glioma stem cells cultured as three-dimensional clusters*. (a,b) Still images from live cell cultures illustrate sphere structure of proneural-GSC1079 cells treated with control media (CMO) (a) and loss of sphere configuration in the presence of endothelial media (CME) (b); Images obtained using Incucyte system over 7 days *in vitro*. (c-h). Sphere forming capacity of pediatric glioma stem cells remains unchanged when cultured in CMO (c, e, g) and HUVEC-conditioned media (CME; d, f, h) after 7 days *in vitro*; Abbreviations: CMO, own conditioned media; CME, conditioned media derived from endothelial cells.

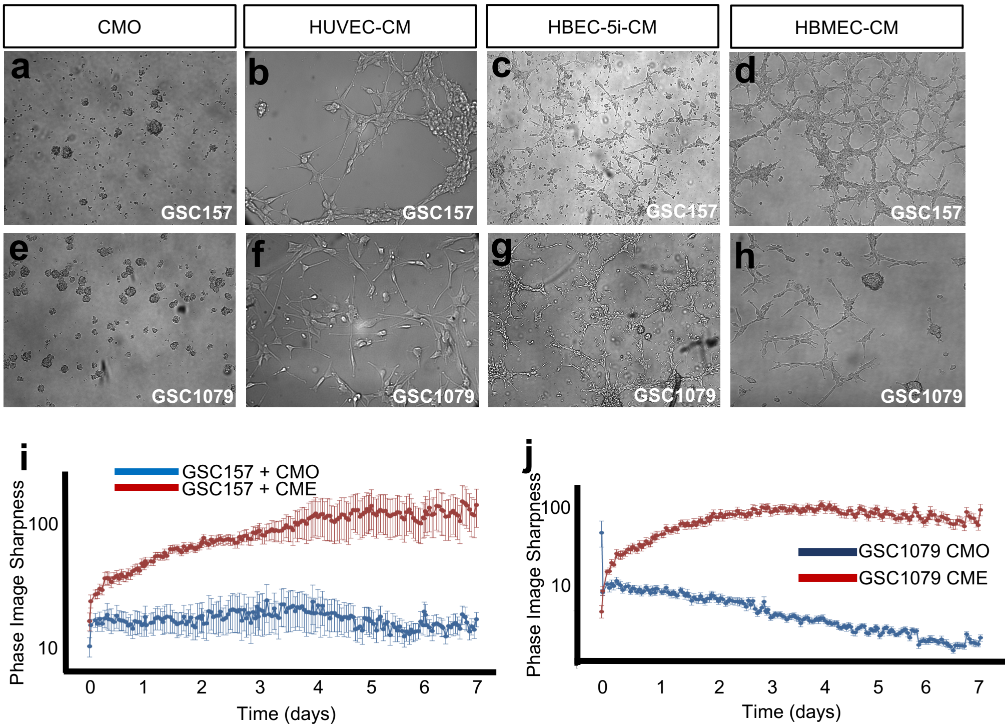

Supplementary figure 4: *Endothelial cell secretome enables transition from sphere formation to adhesive growth coupled with mesenchymal changes in morphology of proneural glioma stem cells.* (a-d) Proneural-GSC157 cells treated with own conditioned media (CMO) (a), or endothelial conditioned media including: HUVEC-CM (b), HBEC5i-CM (c), HBMEC-CM (d) for 7d *in vitro.* (e-h) GSC1079 cells treated with CMO (e), HUVEC-CM (f), HBEC-5i (g), HBMEC-CM (h) for 7d *in vitro.* (i,j) Phase sharpness of GSC157 cells (i) and GSC1079 cells (j) after treatment with CMO (blue line) and HBEC5i conditioned media (CME; red line) measured using Incucyte for 7 days; Abbreviations: CM, conditioned media; CMO, cancer cells' own conditioned media; CME, conditioned media derived from endothelial cells; HUVEC, Human umbilical vein endothelial cells; HBEC5i, immortalized human brain endothelial cells; HBMEC, human brain microvascular endothelial cells.

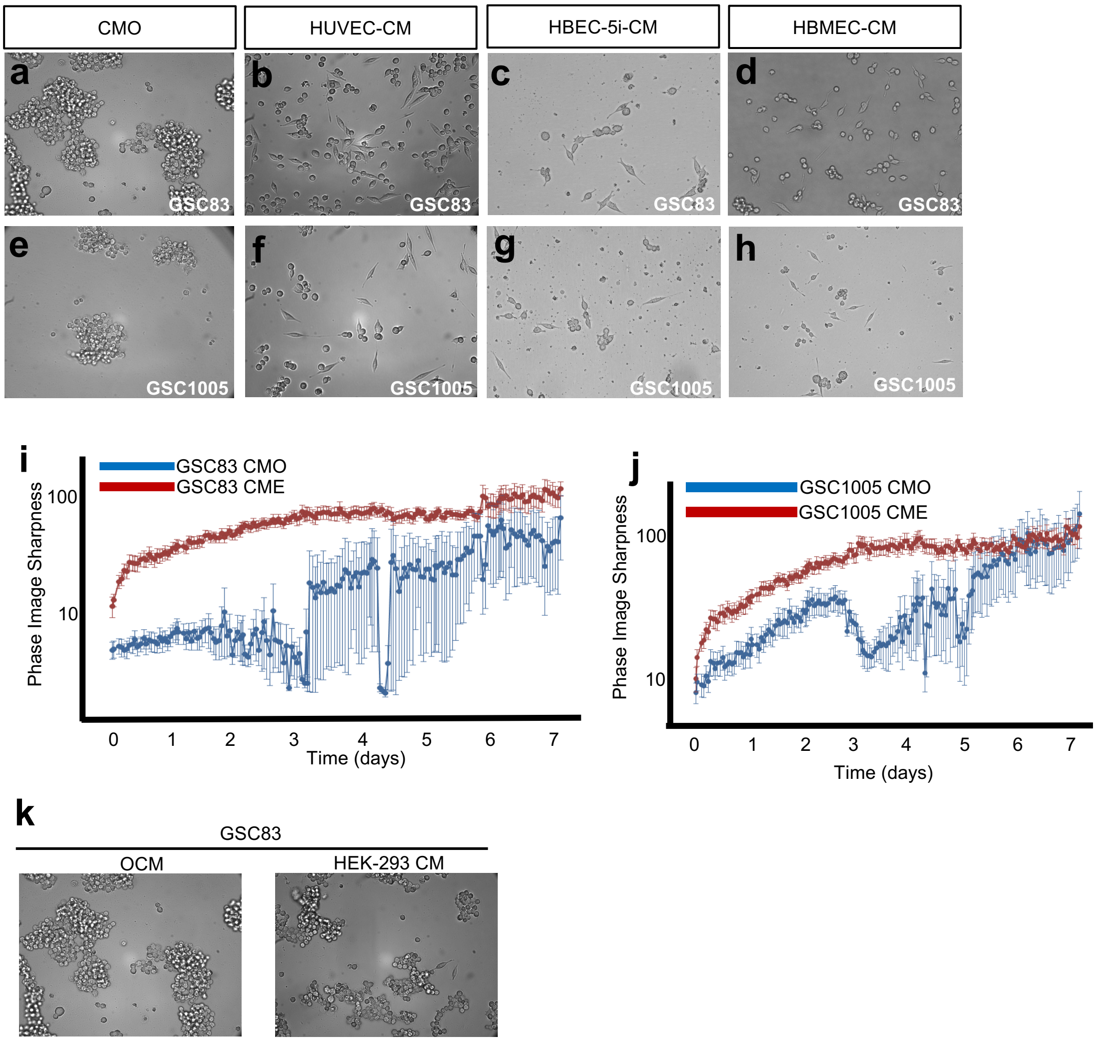

Supplementary figure 5: *Mesenchymal glioma stem cells exhibit modest changes in morphology in the presence of endothelial cell conditioned media.* (a-d) Light microscopy of mesenchymal-GSC83 treated with own conditioned media (CMO) (a), or endothelial conditioned media: HUVEC-CM (b), HBEC-5i (c), HBMEC-CM (d) for 7 days *in vitro.* (e-h) GSC1005 treated with CMO (a), HUVEC-CM (b), HBEC-5i (c), HBMEC-CM (d) for 7 days *in vitro.* (i,j) Phase sharpness of GSC83 (i) and GSC1005 (j) measured using Incucyte for 7 days. (k) Mesenchymal GSC83 treated with OCM and HEK293-CM; Abbreviations: CM, conditioned media; CMO, cancer cells' own conditioned media; CME, conditioned media derived from endothelial cells. HUVEC, Human umbilical vein endothelial cells; HBEC5i, immortalized human brain endothelial cells; HBMEC, human brain microvascular endothelial cells.

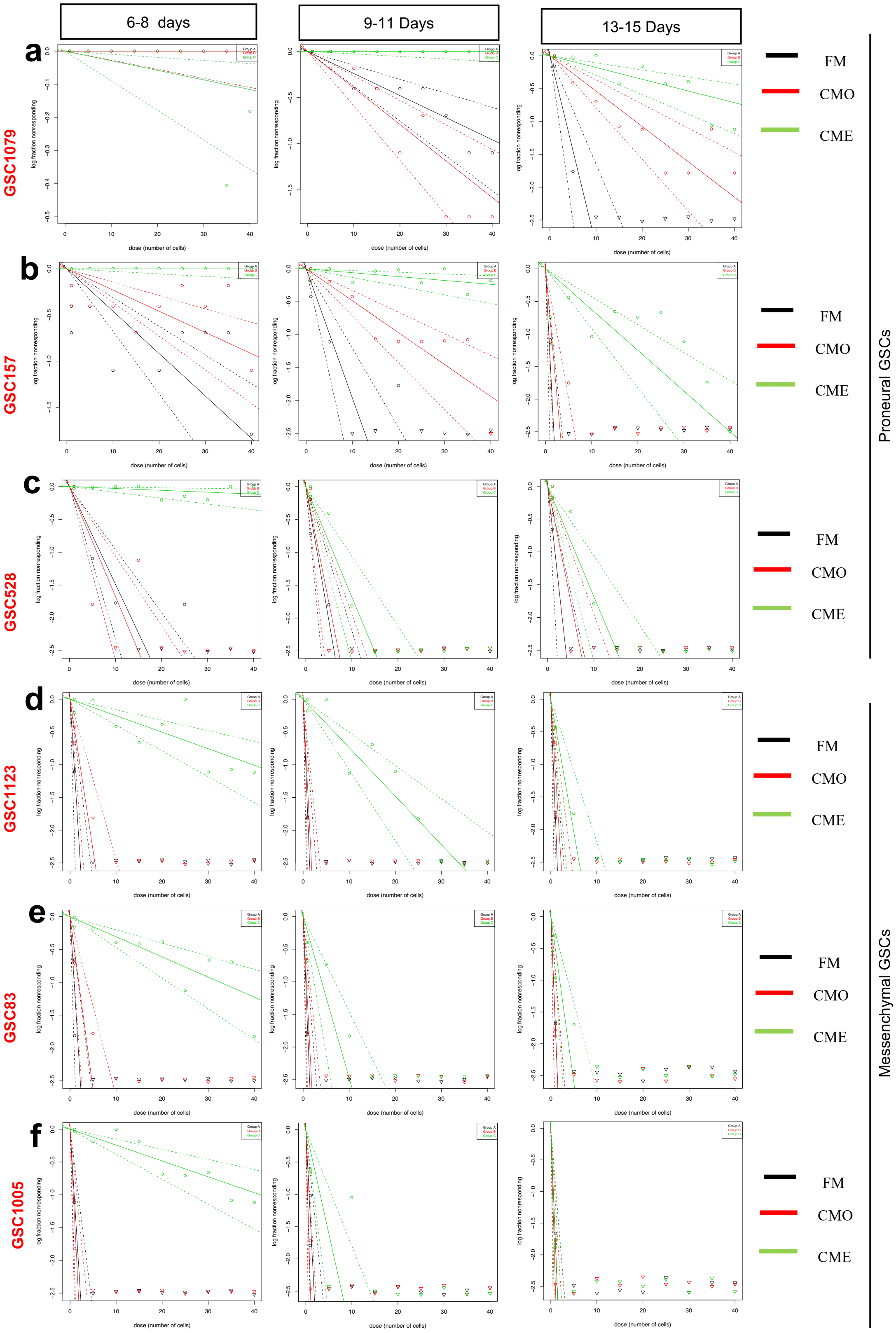
6-8 days
9-11 Days
13-15 Days
a
b
c
d
e
f
GSC1079
GSC157
GSC528
GSC1123
GSC83
GSC1005
FM
CMO
CME
Proneural GSCs
Messenchymal GSCs
log fraction nonresponding
dose (number of cells)
Group A
Group B
Group C

Supplementary figure 6: *ELDA measurements of GSC 'stemness' in the presence of endothelial conditioned media reveals subtype-specific responses.* (a-f) ELDA assays for GSCs treated with fresh media (black line), own conditioned media - CMO (red line) and endothelial conditioned media - CME (green line) for up to 15 days. Proneural-GSCs (a-c) show delayed sphere formation when treated with CME relative to fresh media and CMO, while this effect was rapidly overcome in fast proliferating, more aggressive mesenchymal-GSCs (d-f). Abbreviations: ELDA, extreme limiting dilution analysis; GSC – glioma stem cell, CMO, cancer cells' own conditioned media; CME, conditioned media derived from endothelial cells.

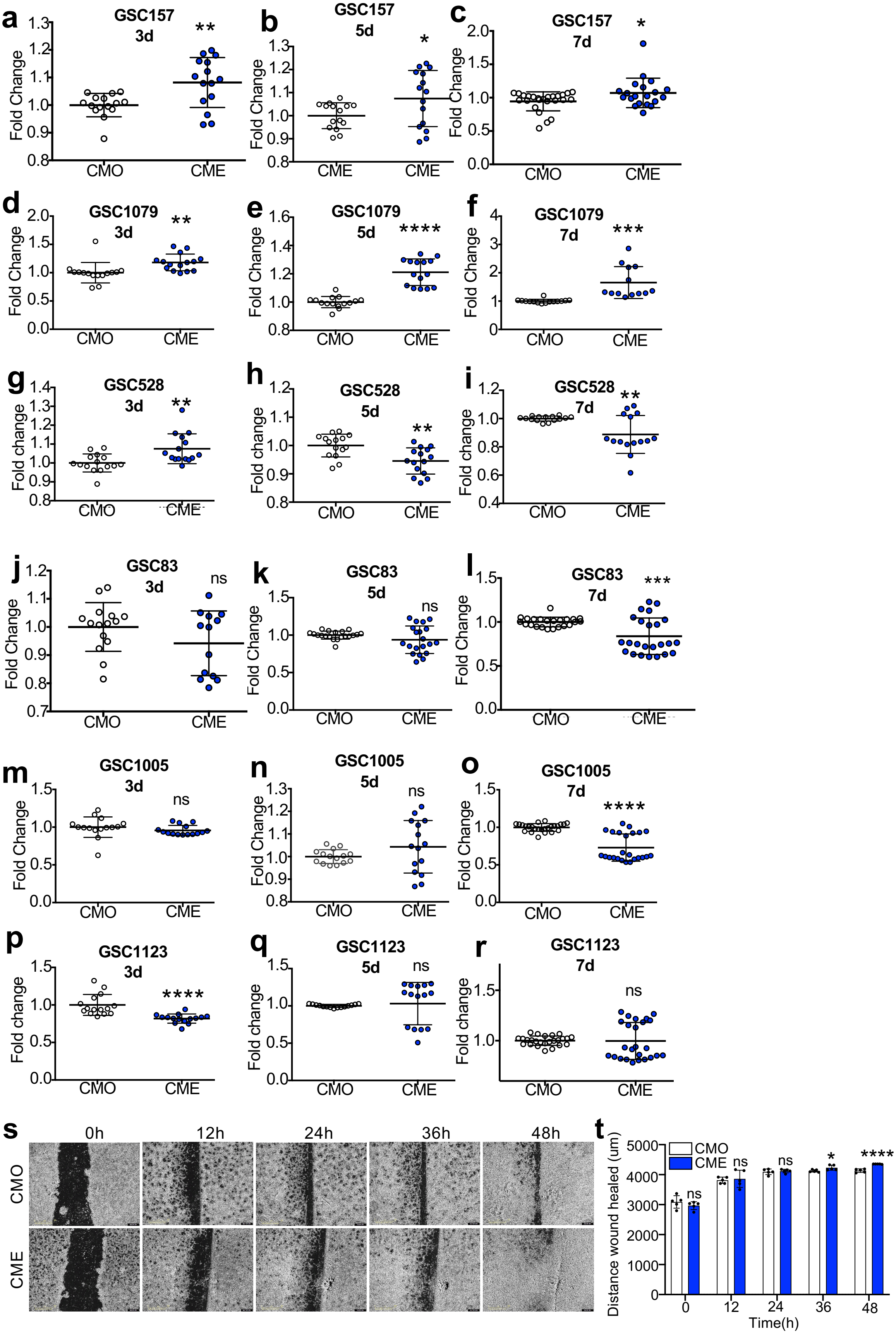

a
GSC157
3d
**
Fold Change
CMO
CME
b
GSC157
5d
*
Fold Change
CME
CME
c
GSC157
7d
*
Fold Change
CMO
CME
d
GSC1079
3d
**
Fold Change
CMO
CME
e
GSC1079
5d
****
Fold Change
CMO
CME
f
GSC1079
7d
***
Fold Change
CMO
CME
g
GSC528
3d
**
Fold change
CMO
CME
h
GSC528
5d
**
Fold change
CMO
CME
i
GSC528
7d
**
Fold change
CMO
CME
j
GSC83
3d
ns
Fold Change
CMO
CME
k
GSC83
5d
ns
Fold Change
CMO
CME
l
GSC83
7d
***
Fold Change
CMO
CME
m
GSC1005
3d
ns
Fold Change
CMO
CME
n
GSC1005
5d
ns
Fold Change
CMO
CME
o
GSC1005
7d
****
Fold Change
CMO
CME
p
GSC1123
3d
****
Fold change
CMO
CME
q
GSC1123
5d
ns
Fold change
CMO
CME
r
GSC1123
7d
ns
Fold change
CMO
CME
s
0h
12h
24h
36h
48h
CMO
CME
t
CMO
CME
ns
ns
ns
*
****
Distance wound healed (um)
Time(h)
0
12
24
36
48

Supplementary figure 7: *Growth, viability and migration of GSCs in the presence of endothelial conditioned media* (a-i) Proneural-GSCs treated with own conditioned media (CMO), or endothelial conditioned media (CME) for 3 days (a, d, g), 5d (b, e, h) and 7 days (c, f, i) tested in cultures of GSC157 (a-c), GSC1079 (d-f) and GSC528 cells (g-i). (j-r) Mesenchymal-GSCs treated with CMO or CME for 3 days (j, m, p), 5 days (k, n, q) and 7 days (l, o, r). Panels depict: GSC83 (j-l), GSC1005 (m-o) and GSC1123 cells (p-r). Each data point represents an individual well tested using MTS. Five biological repeats were conducted per cell line, per time point. (s) Still microphotographs from Incucyte analysis of cell migration in the 'wound healing' assay of proneural-GSC84 cells treated with CMO or CME. (t) Quantification of the distance to which a cell monolayer 'wound' healed in cultures of proneural GSC84 cells treated with CMO or CME. Abbreviations: CMO, cancer cells' own conditioned media; CME, conditioned media derived from endothelial cells.

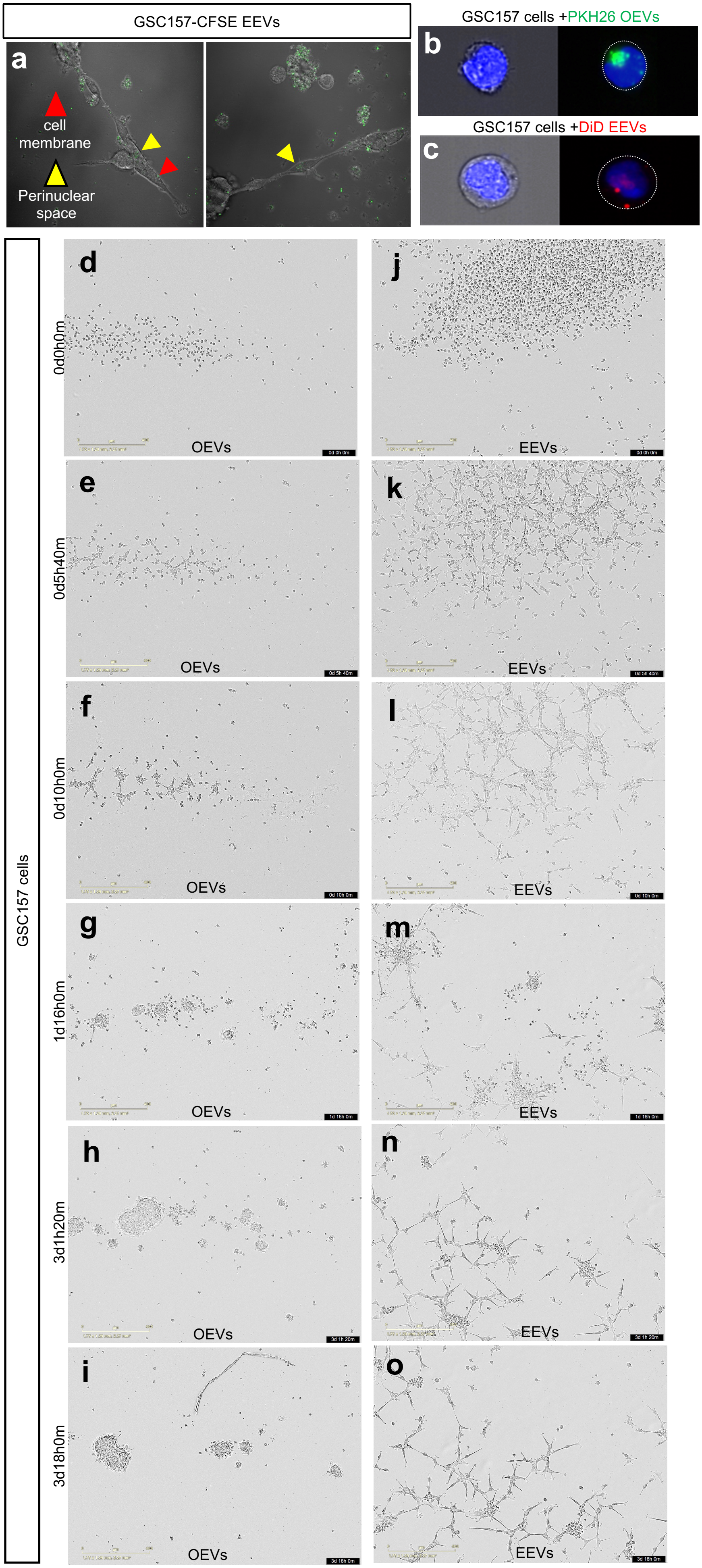
GSC157-CFSE EEVs
a
cell membrane
Perinuclear space
GSC157 cells +PKH26 OEVs
b
GSC157 cells +DiD EEVs
c
GSC157 cells
d
j
0d0h0m
OEVs
EEVs
e
k
0d5h40m
f
l
0d10h0m
g
m
1d16h0m
h
n
3d1h20m
i
o
3d18h0m

Supplementary figure 8: *Distinct cellular changes observed in proneural GSCs after treatment with endothelial cell extracellular vesicles over time.* (a) High resolution confocal microscopy of unlabelled proneural GSC157 cells treated with CFSE labelled EEVs (HUVEC EVs) after 24 hours in culture. Red arrowheads point to EEV related fluorescence in cell membranes, while yellow arrowheads point to EEV fluorescence in perinuclear space within the recipient cell. (b) ImageStream analysis of proneural GSC157 cells labeled with nuclear stain (Nucblue) and treated with PKH26-labelled OEVs. (c) ImageStream analysis of proneural GSC157 cells labeled with Nucblue and treated with DiD labelled EEVs. (d-o) Still images from live cell imaging of GSC157 cells treated with OEVs (d-i) or EEVs (j-o) over 3 days and 18 minutes; Abbreviations: OEVs, cancer cells' own extracellular vesicles; EEV, extracellular vesicles derived from endothelial cells.

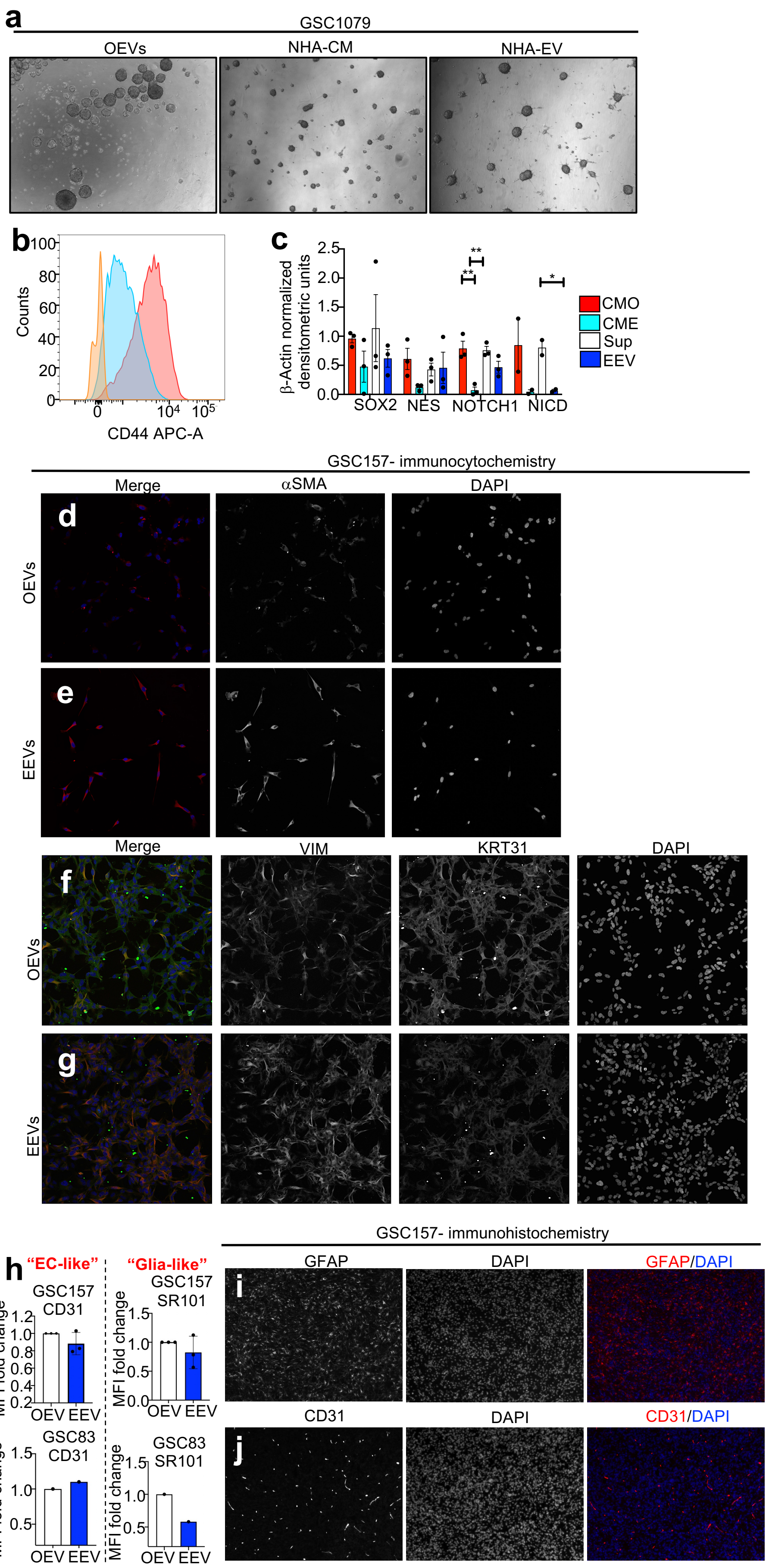
a
GSC1079
OEVs
NHA-CM
NHA-EV
b
Counts
CD44 APC-A
c
β-Actin normalized densitometric units
SOX2
NES
NOTCH1
NICD
CMO
CME
Sup
EEV
GSC157- immunocytochemistry
Merge
αSMA
DAPI
d
e
OEVs
EEVs
Merge
VIM
KRT31
DAPI
f
g
h
"EC-like"
"Glia-like"
GSC157
CD31
GSC157
SR101
GSC83
CD31
GSC83
SR101
MFI fold change
OEV EEV
GSC157- immunohistochemistry
GFAP
DAPI
GFAP/DAPI
i
CD31
DAPI
CD31/DAPI
j

Supplementary figure 9: *Endothelial cell derived extracellular vesicles impose mesenchymal traits upon proneural glioma stem cells.* (a) Proneural-GSC1079 cells treated with own EVs - OEVs, NHA-conditioned media, or NHA-EVs. (b) Flow cytometry for CD44-APC staining of proneural GSC157 cells treated with OEVs and endothelial EVs (EEVs). Orange curve represents IgG control, blue curve represents CD44 levels in proneural GSC157 treated with OEVs, red curve represents CD44 in GSC157 cells treated with EEVs. (c) Densitometric changes in proneural protein markers of proneural GSC157 cells treated with CMO, CME, EEVs and EV-depleted EC-Supernatant (Sup). (d-g) Proneural GSC157 cells were treated with OEVs or EEVs for 7 days and stained for: alpha-smooth muscle actin (αSMA) (d, e), VIM and KRT31 (f,g). (h) FACS analysis of proneural GSC157 cells for endothelial cell marker, CD31, and glial marker, SR101 (top); analogous staining of mesenchymal-GSC83 (bottom). Both cell populations were treated with OEV and HUVEC EVs (EEV) for 3 days *in vitro*. (i,j) Immunofluorescence of mouse brain tissues containing proneural GSC157 intracranial xenografts; staining for GFAP (i) and CD31 (j); Abbreviations: OEVs, cancer cells' own extracellular vesicles; EEV, extracellular vesicles derived from endothelial cells; CMO, cancer cell's own conditioned media; CME endothelial cell conditioned media; NHA, normal human astrocytes; CM, conditioned media; EC, endothelial cells.

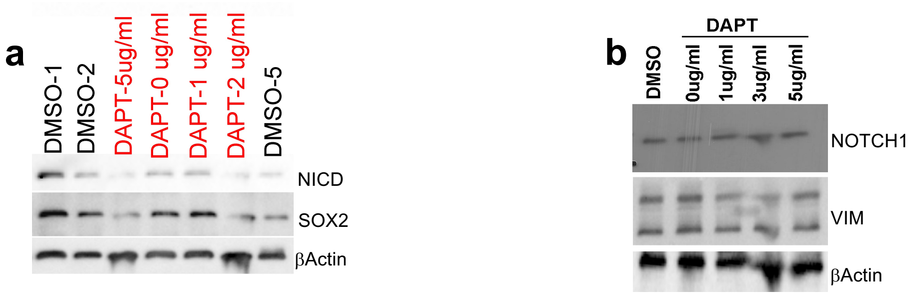

c

Abundance Rank	Protein ID	Protein Name	Avg. weighted spectral counts
61	TGM2	Transglutaminase 2	56.6666667
72	VIM	Vimentin	48.6666667
372	CD44	CD44 antigen	10
1068	KRT18	Keratin, type1 cytoskeletal 18	2

Abundance Rank	Protein ID	Protein Name	Avg. weighted spectral counts
101	MMP2	Type IV collagenase	32.33333333
105	MMP1	Interstitial collagenase	31
660	MMP14	Matrix metaloprotienase 14	4.666666667
1069	MMP11	Stromelysin	2

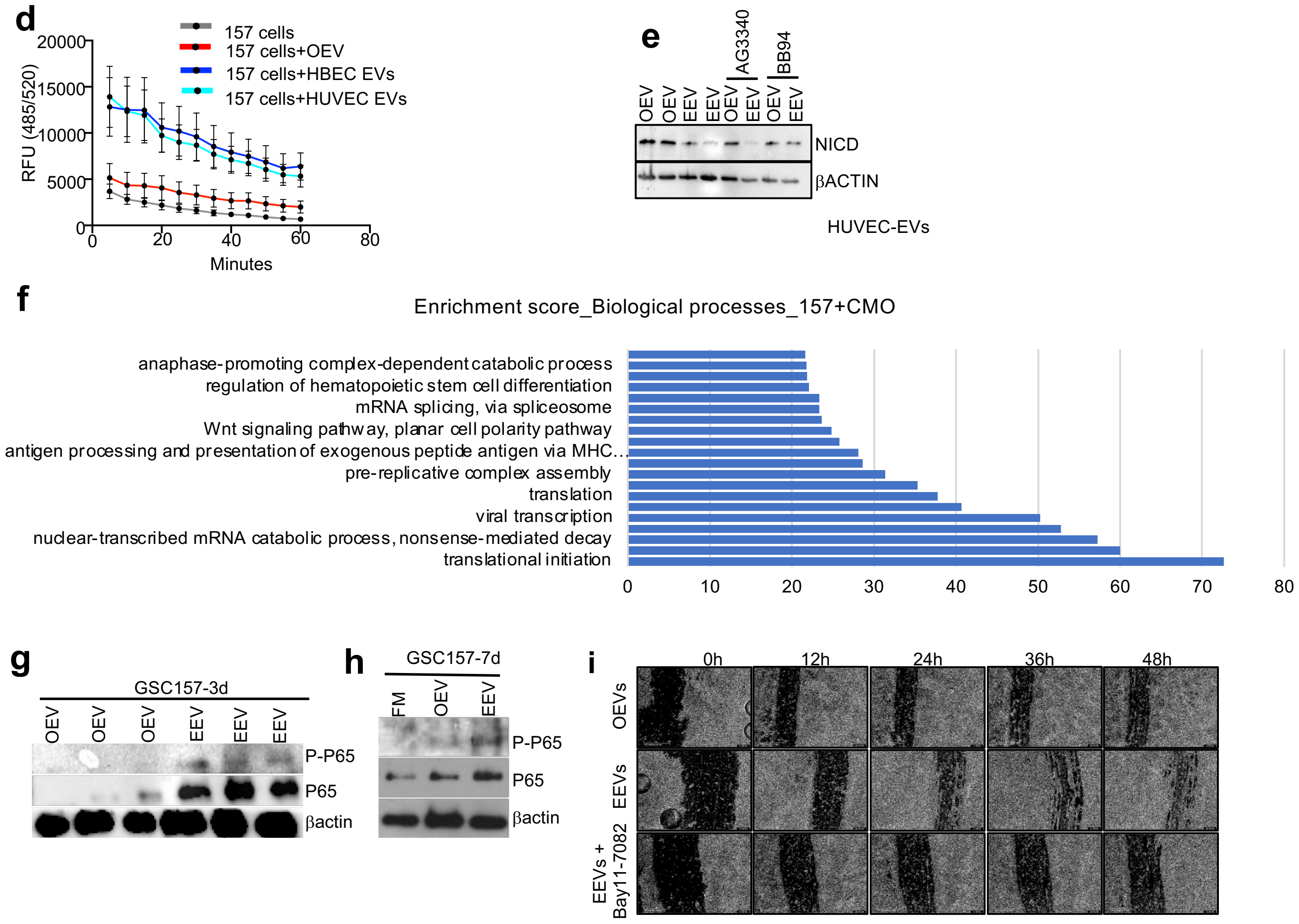

Supplementary figure 10: *NOTCH1 inhibition is insufficient to prevent full mesenchymal reprogramming of proneural GSCs post endothelial extracellular vesicle treatment.* (a) Diminished expression of NICD and SOX2 (proneural markers) in proneural cells (proneural-GSC157) treated with NOTCH1 pathway inhibitor (γ-secretase inhibitor, DAPT), at 0μg/ml, 1μg/ml, 2μg/ml, 5μg/ml or DMSO (vehicle). (b) Unchanged NOTCH1 and VIM expression in proneural-GSC157 treated with DMSO and DAPT at 0μg/ml, 1μg/ml, 2μg/ml, 5μg/ml. (c) Highly enriched mesenchymal proteins (top) and MMPs (bottom) revealed by mass spectrometry of HUVEC EVs (EEVs). (d) MMP activity assay of proneural-GSC157 cells, GSC157 cells treated with OEVs, GSC157 cells treated with HBEC5i EVs, and GSC157 cells treated with HUVEC-EVs over 1 hour (n=3). (e) Expression of NICD following treatment of proneural-GSC157 with OEVs, HUVEC-EVs (EEVs), with or without inhibiting MMP activity using AG3340 and BB94. (f) DAVID analysis of the top pathways enriched in proneural GSC157 treated with CMO. (g, h) Western blots of expression of activated P65 (phospho-P65) and total P65 in GSC157 treated with HBEC5i-EVs (EEVs) for 3 days (g) and with HUVEC-EVs for 7 days (h). (i) Migration (wound healing) assay reveals responses of proneural GSC157 treated with OEVs, EEVs or EEVs+Bay 11-7082 through 48 h; Abbreviations: OEVs, cancer cells' own extracellular vesicles; EEV, extracellular vesicles derived from endothelial cells; CMO, cancer cell's own conditioned media; CME endothelial cell conditioned media; CM, conditioned media; EC, endothelial cells.

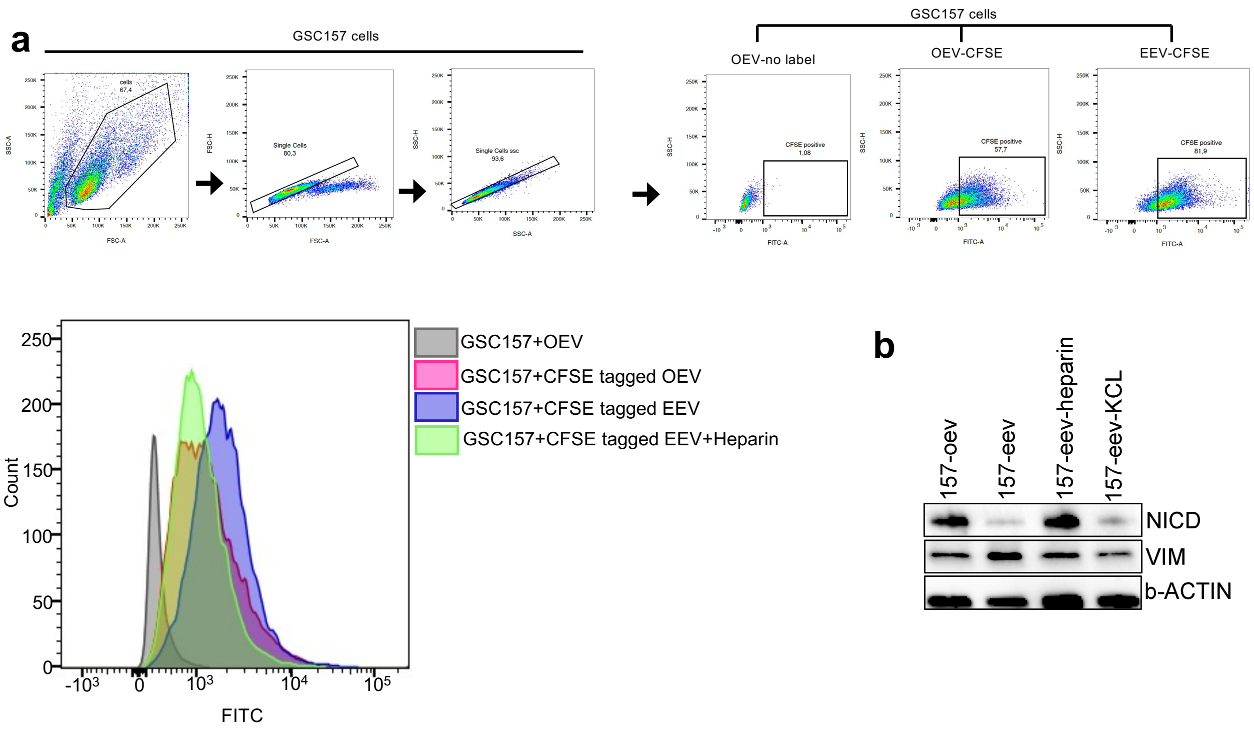

Supplementary figure 11: *Endothelial extracellular vesicles require internalization by proneural GSCs to initiate their reprograming.* (a) Gating strategy used throughout for FACS analysis. Proneural GSC157 cells treated with 30μg of unlabelled own EVs - OEVs, CFSE labeled OEVs, CFSE labeled endothelial EVs (EEVs) and CFSE labeled EEVs in the presence of heparin. (b) Expression of NICD and VIM after treatment of proneural GSC157 with 30μg of unlabelled OEVs, EEVs, EEVs in the presence of heparin, and EEVs after KCL treatment; Abbreviations: OEVs, cancer cells' own extracellular vesicles; EEV, extracellular vesicles derived from endothelial cells.

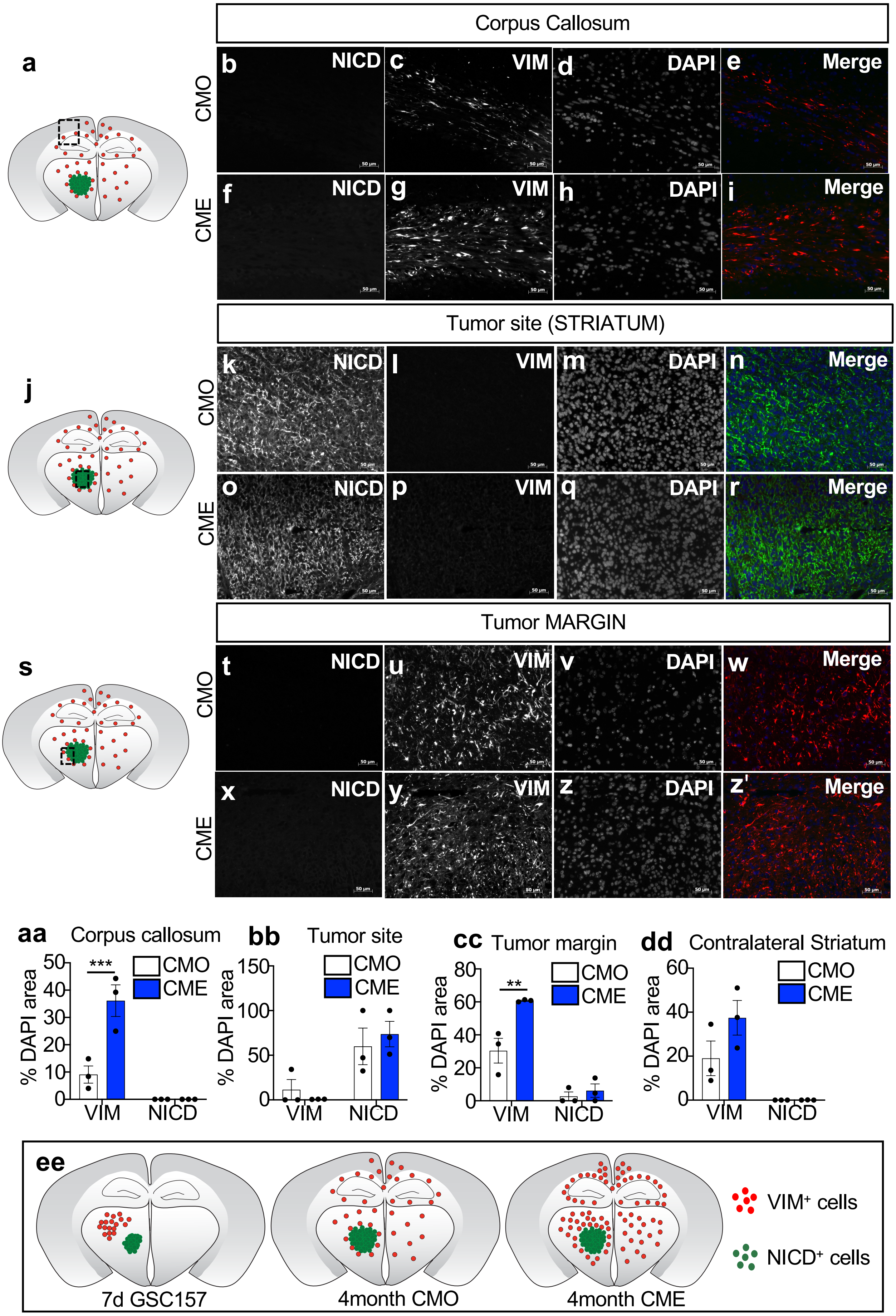

Corpus Callosum
a
CMO
CME
b NICD
c VIM
d DAPI
e Merge
f NICD
g VIM
h DAPI
i Merge
Tumor site (STRIATUM)
j
k NICD
l VIM
m DAPI
n Merge
o NICD
p VIM
q DAPI
r Merge
Tumor MARGIN
s
t NICD
u VIM
v DAPI
w Merge
x NICD
y VIM
z DAPI
z' Merge
50 µm
aa Corpus callosum
bb Tumor site
cc Tumor margin
dd Contralateral Striatum
% DAPI area
CMO
CME
VIM
NICD
***
**
ee
7d GSC157
4month CMO
4month CME
VIM+ cells
NICD+ cells

Supplementary figure 12: *Proneural glioma stem cells exhibit distinct and site-specific NICD and VIM expression profiles and manifest enhanced invasion in vivo following pretreatment with endothelial conditioned media.* (a, j, s) Schematics of the locations of the brain regions studied in the GSC157 intracranial xenografts. (b-z′) Proneural-GSC157 pre-treated with own (CMO) or endothelial conditioned media (CME) and stained for hNICD and hVIM in the corpus callosum (b-i), tumor site (k-r) and at the edge or the margin of the tumor (t- z′). (aa-dd) Percent DAPI area with VIM and NICD expressions in the CMO and CME pretreated GSC157 xenografts in corpus callosum (aa), tumor site (striatum; bb), tumor margin (cc) and striatum in the contralateral hemisphere (dd). The white bars represent CME pretreated GSC157 cells in the different brain regions while the blue bars represent CME pretreated GSC157 cells in the different brain regions. (ee) Summary schematic of murine brain regions with proneural-GSC157 tumors 7 days post implantation, and 4 months after CMO or CME treatment. Red represents VIM^+ tumor cells and green represents $NICD^+$ tumor cells; Abbreviations: CMO, own conditioned media; CME, conditioned media derived from endothelial cells.
