# Supplementary material for: Angiocrine extracellular vesicles impose mesenchymal reprogramming upon proneural glioma stem cells
Source: Nat Commun. 2022 Sep 19;13:5494. doi: 10.1038/s41467-022-33235-7 (PMC9485157; doi:10.1038/s41467-022-33235-7)
Supplement: Supplementary file 2 — Description of Additional Supplementary Files [file 41467_2022_33235_MOESM2_ESM.pdf]

Description of Additional Supplementary Files

File Name: Supplementary Movie 1
Description: Cancer cell incubation in their own conditioned media allows sphere formation by proneural glioma stem cells (GSC1079).

File Name: Supplementary Movie 2
Description: Cancer cell incubation in endothelial conditioned media disrupts sphere formation by proneural glioma stem cells (GSC1079).

File Name: Supplementary Movie 3
Description: Cancer cell incubation with their own extracellular vesicles allows sphere formation by proneural glioma stem cells (GSC157).

File Name: Supplementary Movie 4
Description: Cancer cell incubation with endothelial extracellular vesicles disrupts sphere formation by proneural glioma stem cells (GSC157).
