# Supplementary material for: Angiocrine extracellular vesicles impose mesenchymal reprogramming upon proneural glioma stem cells
Source: Nat Commun. 2022 Sep 19;13:5494. doi: 10.1038/s41467-022-33235-7 (PMC9485157; doi:10.1038/s41467-022-33235-7)
Supplement: Supplementary file 7 — Reporting Summary [file 41467_2022_33235_MOESM7_ESM.pdf]

nature portfolio

Corresponding author(s): Janusz Rak

Last updated by author(s): Aug 18, 2022

Reporting Summary

Nature Portfolio wishes to improve the reproducibility of the work that we publish. This form provides structure for consistency and transparency in reporting. For further information on Nature Portfolio policies, see our Editorial Policies and the Editorial Policy Checklist.

Statistics

For all statistical analyses, confirm that the following items are present in the figure legend, table legend, main text, or Methods section.

n/a	Confirmed	
☐	☒	The exact sample size (*n*) for each experimental group/condition, given as a discrete number and unit of measurement
☐	☒	A statement on whether measurements were taken from distinct samples or whether the same sample was measured repeatedly
☐	☒	The statistical test(s) used AND whether they are one- or two-sided<br>*Only common tests should be described solely by name; describe more complex techniques in the Methods section.*
☒	☐	A description of all covariates tested
☐	☒	A description of any assumptions or corrections, such as tests of normality and adjustment for multiple comparisons
☐	☒	A full description of the statistical parameters including central tendency (e.g. means) or other basic estimates (e.g. regression coefficient) AND variation (e.g. standard deviation) or associated estimates of uncertainty (e.g. confidence intervals)
☐	☒	For null hypothesis testing, the test statistic (e.g. *F*, *t*, *r*) with confidence intervals, effect sizes, degrees of freedom and *P* value noted<br>*Give P values as exact values whenever suitable.*
☒	☐	For Bayesian analysis, information on the choice of priors and Markov chain Monte Carlo settings
☒	☐	For hierarchical and complex designs, identification of the appropriate level for tests and full reporting of outcomes
☒	☐	Estimates of effect sizes (e.g. Cohen's *d*, Pearson's *r*), indicating how they were calculated

Our web collection on statistics for biologists contains articles on many of the points above.

Software and code

Policy information about availability of computer code

Data collection: Thermo Orbitrap Fusion mass spectrometer operating at 120,000 resolution (FWHM in MS1) with HCD sequencing (15,000 resolution).

Data analysis: Prism 9, The raw data were converted into *.mgf format (Mascot generic format) for searching using the Mascot 2.6.2 search engine (Matrix Science) against Human Uniprot sequences (2020).The database search results were loaded onto Scaffold Q+ Scaffold_4.9.0 (Proteome Sciences) for statistical treatment and data visualization, DAVID bioinformatics resource 6.8 (https://david.ncifcrf.gov) for the proteomics data generated.

For flow cytometry, FlowJo software 10.7.1 was used.

For manuscripts utilizing custom algorithms or software that are central to the research but not yet described in published literature, software must be made available to editors and reviewers. We strongly encourage code deposition in a community repository (e.g. GitHub). See the Nature Portfolio guidelines for submitting code & software for further information.

Data

Policy information about availability of data

All manuscripts must include a data availability statement. This statement should provide the following information, where applicable:

- Accession codes, unique identifiers, or web links for publicly available datasets
- A description of any restrictions on data availability
- For clinical datasets or third party data, please ensure that the statement adheres to our policy

The mass spectrometry proteomics data have been deposited to the ProteomeXchange Consortium via the PRIDE [1] partner repository with the dataset identifier PXD034819.

Field-specific reporting

Please select the one below that is the best fit for your research. If you are not sure, read the appropriate sections before making your selection.

☒ Life sciences ☐ Behavioural & social sciences ☐ Ecological, evolutionary & environmental sciences

For a reference copy of the document with all sections, see nature.com/documents/nr-reporting-summary-flat.pdf

Life sciences study design

All studies must disclose on these points even when the disclosure is negative.

Sample size

The sample size was equal or larger than 3 for most experiments, while for intra-cranial injections and tumour staining the sample size was equal or larger than 5. No method was used to calculate the sample size as minimum sample size used per experiment was 3 per group which was increased to 5 or more depending upon availability of resources. 50-50% distribution between male and female mice was used. We applied standard statistical methods and all data referred to as significant has p<0.05 for all the experimental quantification and Analytical/ Molecular techniques

Data exclusions

No data were excluded from the analysis.

Replication

We replicated and repeated all our experiments a minimum of three times in order to allow statistical quantification.

Randomization

All mice/ experiments were randomly assigned to treated or no-treated.

Blinding

The investigators were not blinded to the design of the study due to the experimental conditions. However during injections of tumor cells the mouse surgeon was blinded to the experimental conditions. Mice were grouped according to the vial of cells from which mice were injected which were labeled with a specific code blinded to the observer. All images were taken as per the group. For sex distribution, mice were randomly taken at surgery to have equal numbers of mice for each sex. Mice were housed individually post surgery. Measures were taken to objectively and repeatedly assess the results, including replicating the visual observation with instrumental measurements (e.g. exCelligence).

For all in vitro experiments, the labeled plate lids were replaced for unlabeled lids to avoid bias during data collection. The analysis was performed using the labeled lids per experiment.

Reporting for specific materials, systems and methods

We require information from authors about some types of materials, experimental systems and methods used in many studies. Here, indicate whether each material, system or method listed is relevant to your study. If you are not sure if a list item applies to your research, read the appropriate section before selecting a response.

Materials & experimental systems

n/a	Involved in the study	
☐	☒	Antibodies
☐	☒	Eukaryotic cell lines
☒	☐	Palaeontology and archaeology
☐	☒	Animals and other organisms
☒	☐	Human research participants
☒	☐	Clinical data
☒	☐	Dual use research of concern

Methods

n/a	Involved in the study	
☒	☐	ChIP-seq
☐	☒	Flow cytometry
☒	☐	MRI-based neuroimaging

Antibodies

Antibodies used

Primary antibody dilutions used were as per the data-sheet provided per antibody:
rabbit anti-CD63 (ab134045, Abcam, Lot#GR3178123-2),
rabbit anti-CD9 (ab92726, Abcam, Lot#GR269281-11),
rabbit anti-CD81 (ab155760 Abcam, Lot#GR182632-11),
rabbit anti-BIP (3183S, Cell Signalling, Lot#4),
rabbit anti-NOTCH1 (4380, cell signalling, Lot#4),
rabbit anti-SOX2 (ab93689, Abcam, Lot#GR3246237-14),
rabbit anti-NES (ab105389, Abcam, Lot#GR-3224771-41),
rabbit anti-TGM2 (3557S, Cell Signalling, Lot#3),
rabbit anti-NICD (07-1231, Millipore, Lot#3506695),
goat anti-VIM (AF2105-SP, R&D, Lot#KRZ0917101),
rabbit anti-P65 (8242T, cell signalling, Lot#16),
rabbit anti-pP65 (3033S, cell signalling, Lot#17)
mouse anti-b-actin (A1978, Sigma)

rabbit anti-CD31 (SAB5600061, Sigma, Lot#S0802450),
mouse anti-NES (MAB1259)

Secondary antibodies (1:500):
HRP-anti rabbit (7074 Cell Signalling, Lot#29)
HRP-anti mouse (170-6516 Biorad, Lot#5680)
Goat anti-Rabbit IgG Alexa Fluor 488 (A-11034 Invitrogen, Lot#018207)
Donkey anti-Goat IgG Alexa Fluor Plus 594 (A32758 Invitrogen, Lot#T1271730A)

Validation

Validation of CD63, CD9, CD81 and BIP:

Manufacturers validated:
CD63 using human melanoma cell, Hs68 and T24 whole cell lysates;
CD9 using HeLa cell line;
CD81 using K562 whole cell lysate;
BIP using several cell lines.

In manuscript:
validated using GSC and HUVEC cell and EV samples on the western blot using dilutions ranging between 1:500-1:1000.

Validation of NOTCH1, SOX2, NES and NICD antibodies:

Manufacturers validated:
NOTCH1 using extracts from HPB-ALL and Molt4;
SOX2 using human breast adenocarcinoma cell line lysate;
NES using HeLa cell lysate;
NICD using A431 and HeLa cel lysates, and IHC using a lipoma tissue section.
NOTCH1 and NICD were used at 1:1000 dilution,
SOX2 and NES were used at 1:200 dilution.

In manuscript:
Performed on proneural glioma stem cell lines (157 and 1079) known to express high levels of these proteins.

Validation of TGM2 and VIM:

Manufactures validated:
TGM2 and VIM using various cell lines and IHC for VIM using human skin tissue.
Dilution used was 1:1000 for both the antibodies.

In manuscript:
using mesenchymal cell lines 83 and 1005 which are known to express these proteins at a high levels.

Manufactures validated P65 and p-P65 using various cell lines including HeLa and NIH3T3.
Dilution used was 1:1000 for both the antibodies.

b-actin was validated using all cell lines in the study. Manufacturers validated b-actin using several cell lines. It was used in 1:1000 dilution.

Eukaryotic cell lines

Policy information about cell lines

Cell line source(s)

Cell lines used through out the study (GSC157, GSC1079, GSC84, GSC528, GSC83, GSC1005 and GSC1123) were provided by Dr. Ichiro Nakano from the Ohio State University, USA.

HUVEC (Catalog No. PCS-100-010), immortalized HBEC-5i (Catalog No. CRL-3245) and HEK-293 (Catalog No. CRL-1573) were obtained from ATCC and cultured in the recommended media or as indicated. Primary human brain microvascular endothelial cells (Catalog No. H-6023) were obtained from Cell Biologics.
Patient derived glioma stem cell, both proneural (GSC157; GSC1079; GSC528; GSC84) and mesenchymal (GSC83; GSC1005; GSC1123) subtype were isolated from male or female patients with Glioblastoma tumours, as previously described (Mao et al. 2013).

pLV-CMV-LoxP-DsRed-LoxP-eGFP (plasmid 65726, addgene) lentiviral vector was transduced in the GSC157 cells.
pLM-CMV-R-Cre (plasmid 27546, addgene) lentiviral vector was transduced into HUVECs.

Authentication

Cell lines used were either obtained from commercial sources and validated by suppliers, or patient derived glioma stem cells provided by Dr. Ichiro Nakano. The gene and protein expression studies are periodically performed in our lab (PMID: 29016925, this paper) confirm the identity of cell lines as published (PMID: 23650391) by RNA seq, Western blot, immunostaining for pluripotency markers and determining differentiation capacity.
The transduced cell lines in the laboratory were validated by FACS.

	Authentication procedures used for cell lines used: For patient derived glioma stem cells protein and mRNA analysis were compared to profiles obtained at the time of initial isolation (PMID: 23650391) were used to authenticate individual cell lines. As these isolates are unique no fingerprinting methods are presently available to perform standard authentication.
Mycoplasma contamination	The cell lines were routinely checked and tested negative for mycoplasma.
Commonly misidentified lines (See ICLAC register)	No commonly misidentified lines were used in the study.

Animals and other organisms

Policy information about studies involving animals; ARRIVE guidelines recommended for reporting animal research

Laboratory animals	All mice used in this study were obtained from the Jackson Laboratory. NOD scid IL2Rgamma-null (NSG) transgenic mice were chosen at 3 months of age for intra-cranial injections. Equal number of males and females were used through out the study. For Aortic ring assay, C57bl/6 mice at 2 months was used. All mice were maintained at MUHC RI and McGill University animal care facility, under 12 hours of light/12 hours of dark cycle.
Wild animals	No wild animals were used in this study.
Field-collected samples	No field collected samples were used in this study.
Ethics oversight	All procedures involving animals were performed in accordance with the guidelines of the Canadian Council of Animal Care and the Animal Utilization Protocols, approved by the Institutional Animal Care Committee at the McGill University Health Centre Research Institute and McGill University (Protocol #5200).

Note that full information on the approval of the study protocol must also be provided in the manuscript.

Flow Cytometry

Plots

Confirm that:

☒ The axis labels state the marker and fluorochrome used (e.g. CD4-FITC).

☒ The axis scales are clearly visible. Include numbers along axes only for bottom left plot of group (a 'group' is an analysis of identical markers).

☒ All plots are contour plots with outliers or pseudocolor plots.

☒ A numerical value for number of cells or percentage (with statistics) is provided.

Methodology

Sample preparation	GSC157 cultures (glioblastoma stem cell received from Dr. Ichiro Nakano) were treated with own conditioned media, huvec cell conditioned media, own extracellular vesicles or HUVEC cell extracellular vesicles for 7 days. Next, the treated cells were collected, deprived of cell debris by spinning at 300 xg, treated with strip cell (Catalog No. 325-055-EL, Wisent) to break spheres then stained for APC conjugated CD44 (338805, Bio Legend) or IgG control. The cells were washed and maintained as single cells prior to flow cytometry.<br><br>For CFSE EV uptake experiment, purified EVs were stained with CFSE (Catalog No. C34554, ThermoFisher). GSC157 cells were treated with CFSE-labelled and non-CFSE labelled EVs. Following the treatment, the cells were collected, deprived of cell debris and ensured single cell suspension.<br><br>Flow cytometry (BD LSR Fortessa) was used to collect data which was analyzed using FlowJo software 10.7.1.
Instrument	BD LSRFortessa Cell Analyser cat#647800. Model number 647800L6. Made in USA.
Software	FlowJo software 10.7.1
Cell population abundance	For the CD44-APC flow cytometry analysis, gated cell populations were analyzed at 10,000 single cell populations in all conditions.<br><br>Cell purity was marked using western blots for NOTCH1, NES and SOX2 (proneural glioma stemccells) and TGM2, VIM (mesenchymal glioma stem cells). CD44 is expressed abundantly in mesenchymal glioma stem cells and not so much in the proneural glioma stem cells. Using flow cytometry, subtype cell purity is confirmed.<br><br>FSC-A/SSC-A gates were used to gate the cell population. Separation of singlets from doublets (FSC-H/FSC-A and SSC-H/SSC-A) followed by demarcating live cells using viability dye (sytox blue, invitrogen S34857). This was used to determine the purity of samples.
Gating strategy	FSC-A/SSA gates were used to gate the cell population followed by separation of singlets from doublets (FSC-H/FSC-A and SSC-H/SSC-A). Voltages for the different lasers used were set to ensure that the positive cell populations started after laser-Area 10e3 (for example, the cells under the curve in APC-A starting at 10e3, indicated cells positive for APC). Cell populations

on the left of 10e3 were considered negative for the marker of interest. These voltages were set up based on the cells stained with IgG control to separate the positive from negative populations.

☒ Tick this box to confirm that a figure exemplifying the gating strategy is provided in the Supplementary Information.
