# Supplementary figures and images for: Angiocrine extracellular vesicles impose mesenchymal reprogramming upon proneural glioma stem cells
Source: Nat Commun. 2022 Sep 19;13:5494. doi: 10.1038/s41467-022-33235-7 (PMC9485157; doi:10.1038/s41467-022-33235-7)

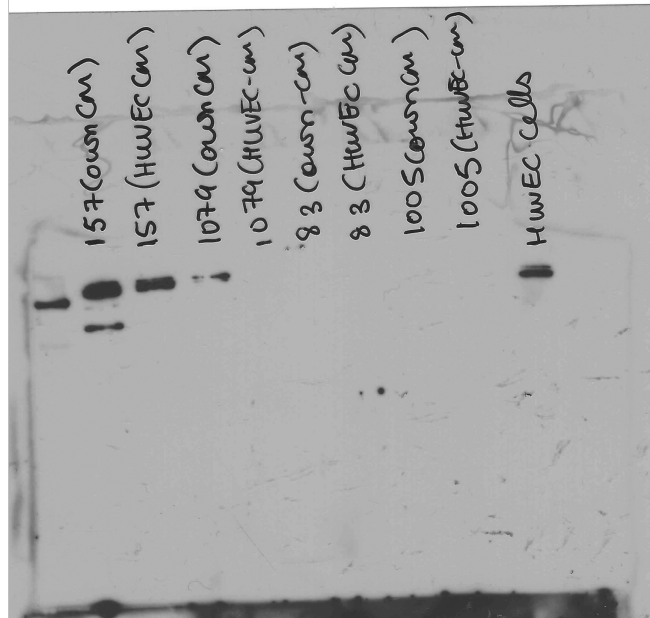

NES

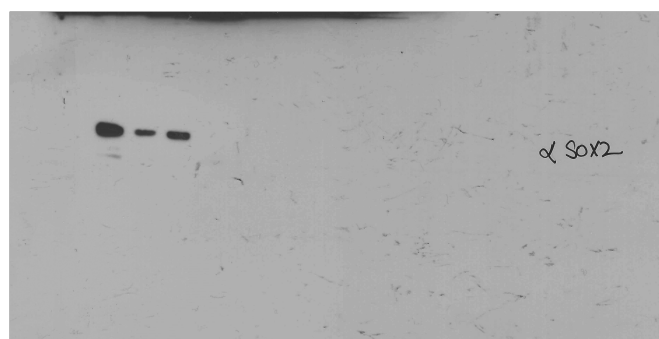

SOX2

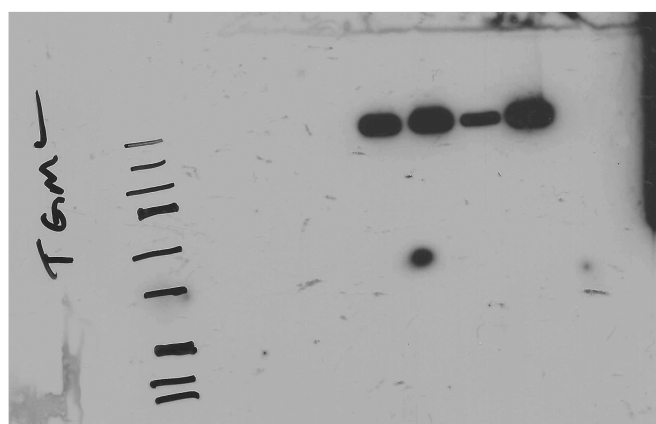

TGM2

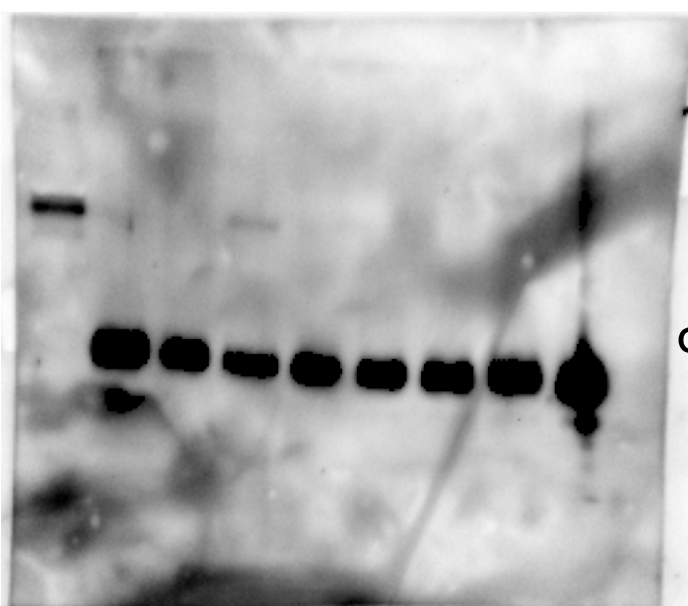

GAPDH

Supplement: Supplementary file 8 — Source Data [file 41467_2022_33235_MOESM8_ESM.zip › SOURCE DATA-92/figure 3-source data.pdf]

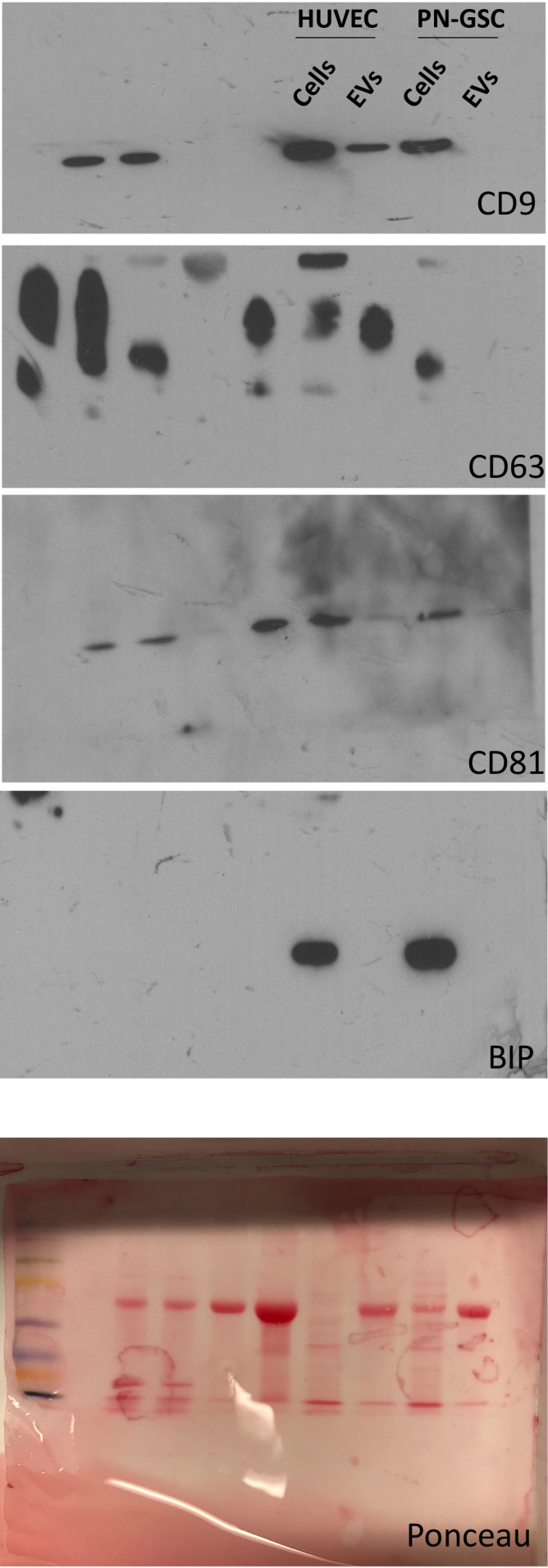

FIGURE 4-source data

Supplement: Supplementary file 8 — Source Data [file 41467_2022_33235_MOESM8_ESM.zip › SOURCE DATA-92/figure 4c-source data.pdf]

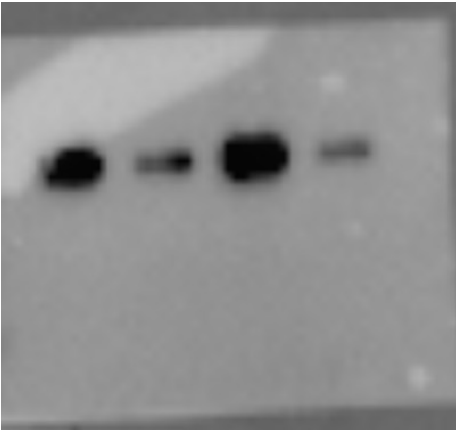

SOX2

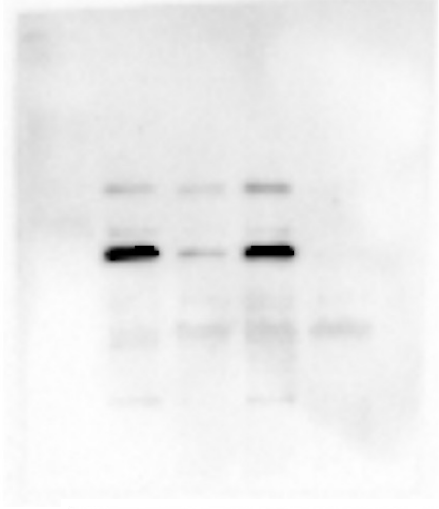

NICD

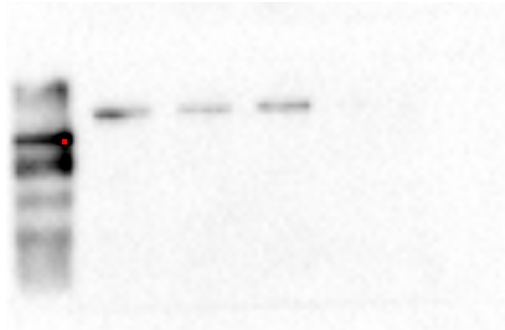

NES

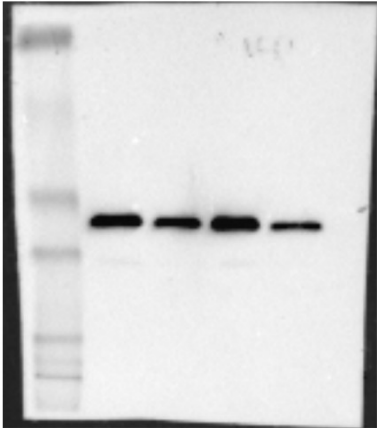

$\beta$ ACTIN

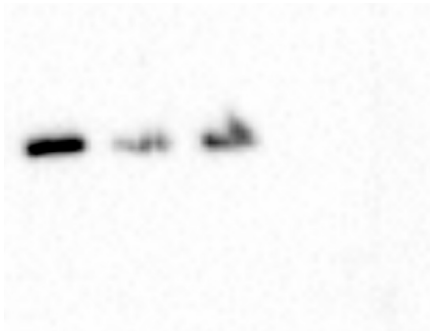

NOTCH1

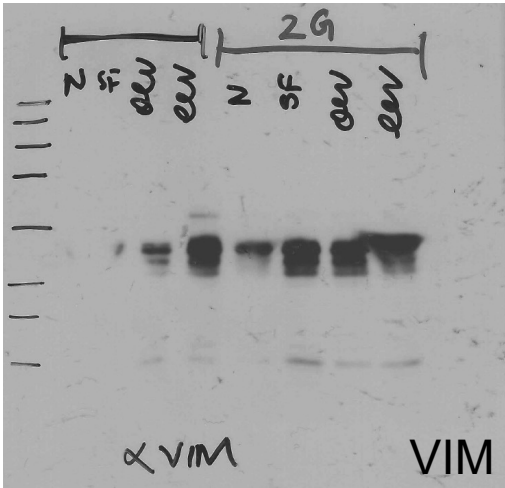

$\alpha$ VIM

VIM

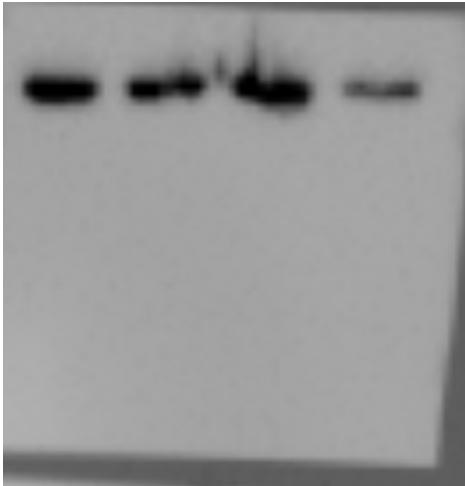

$\beta$ ACTIN

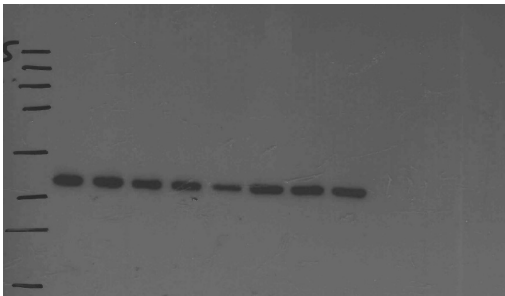

$\beta$ ACTIN

FIGURE 4h-source data

Supplement: Supplementary file 8 — Source Data [file 41467_2022_33235_MOESM8_ESM.zip › SOURCE DATA-92/figure 4h-source data.pdf]

AG3340 BB94

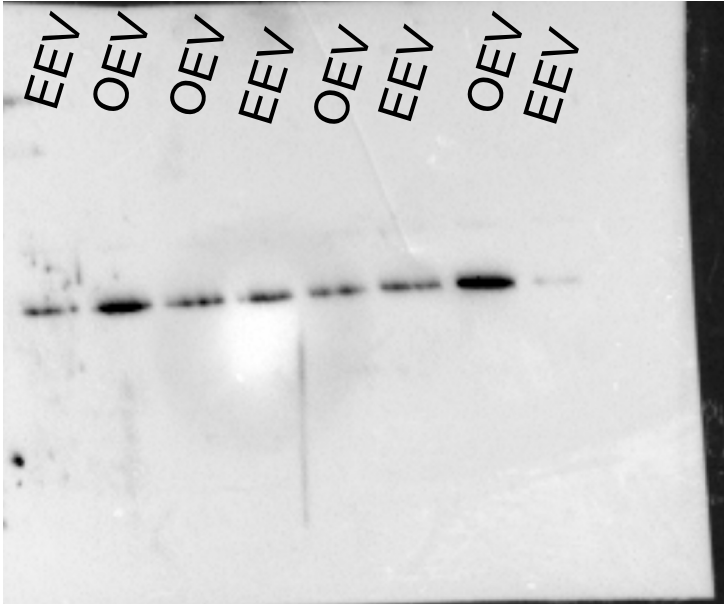

NICD

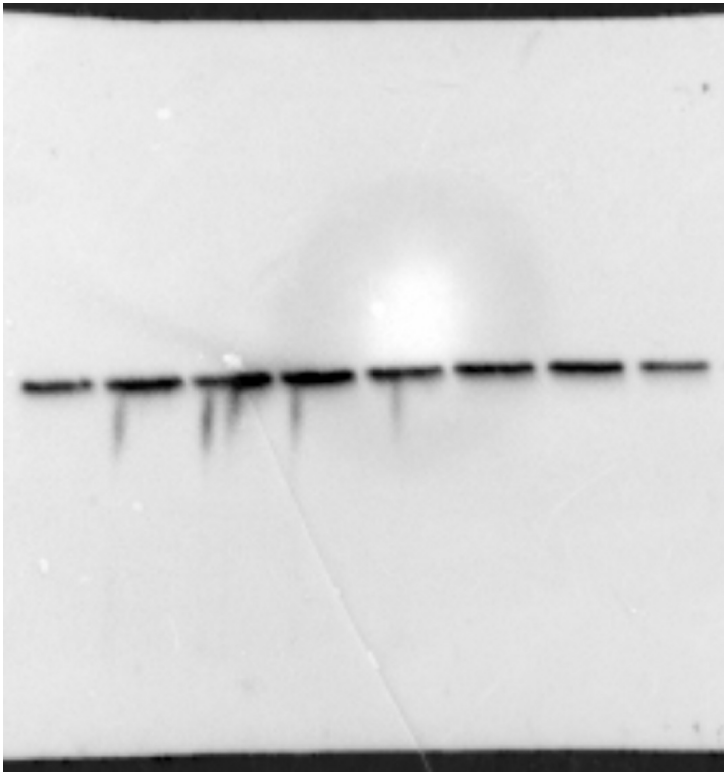

$\beta$ ACTIN

Supplement: Supplementary file 8 — Source Data [file 41467_2022_33235_MOESM8_ESM.zip › SOURCE DATA-92/figure 5f-source data.pdf]

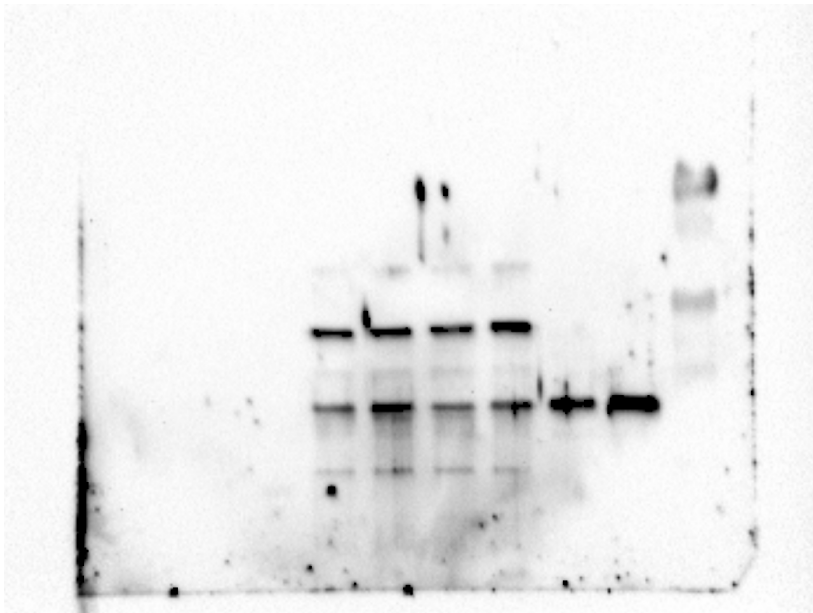

NICD

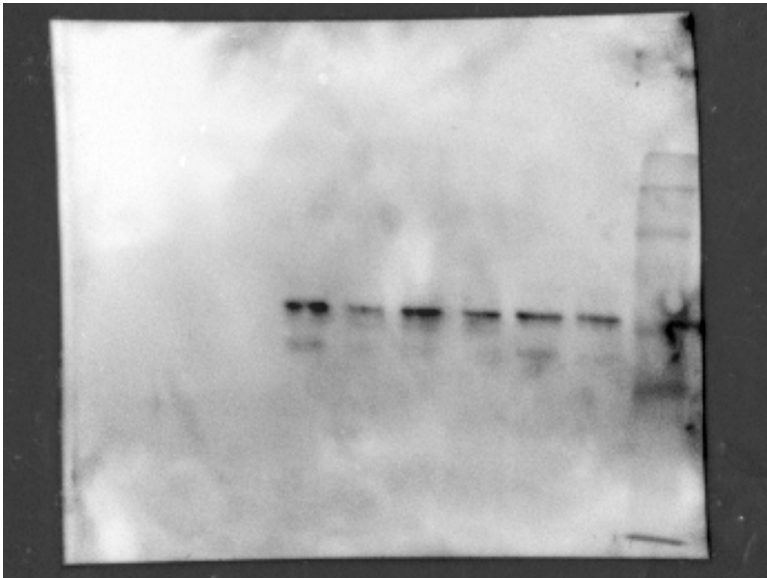

VIM

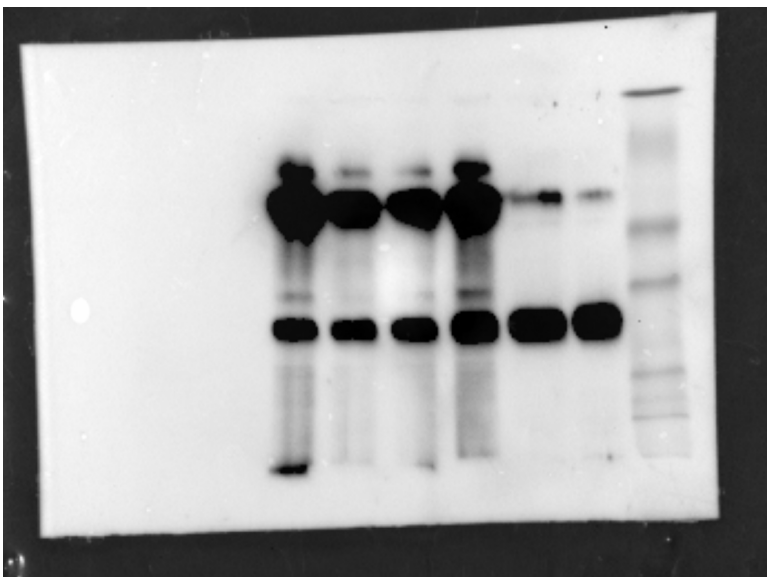

GAPDH

Supplement: Supplementary file 8 — Source Data [file 41467_2022_33235_MOESM8_ESM.zip › SOURCE DATA-92/figure 5h-source data.pdf]

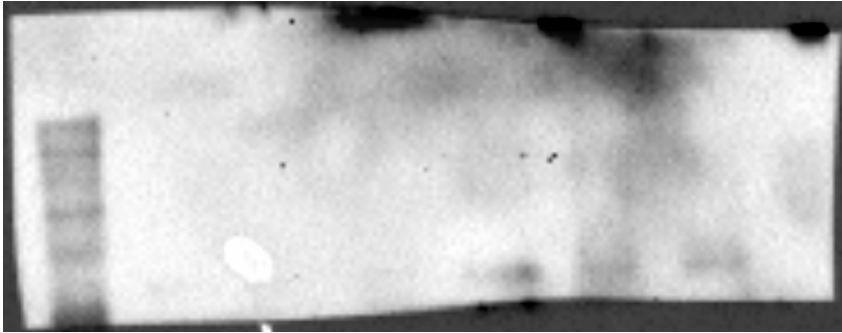

p-P65

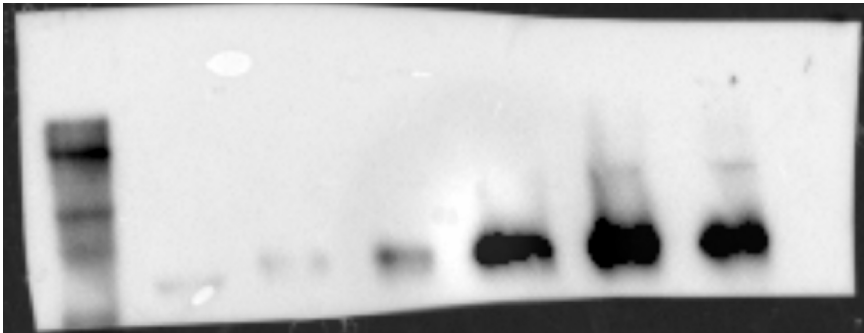

P65

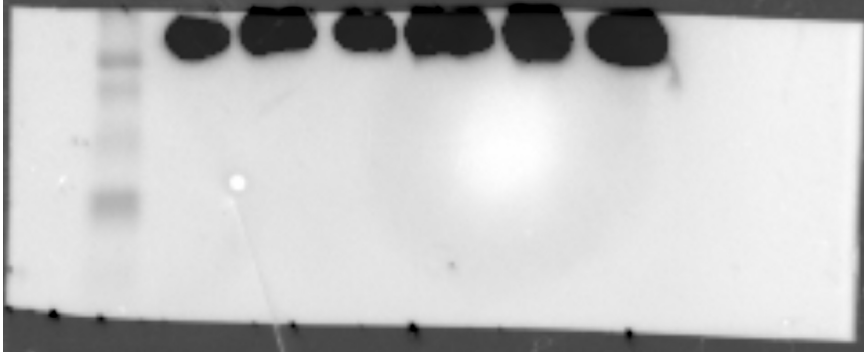

$\beta$ ACTIN

Supplement: Supplementary file 8 — Source Data [file 41467_2022_33235_MOESM8_ESM.zip › SOURCE DATA-92/figure 5jk-source data.pdf]

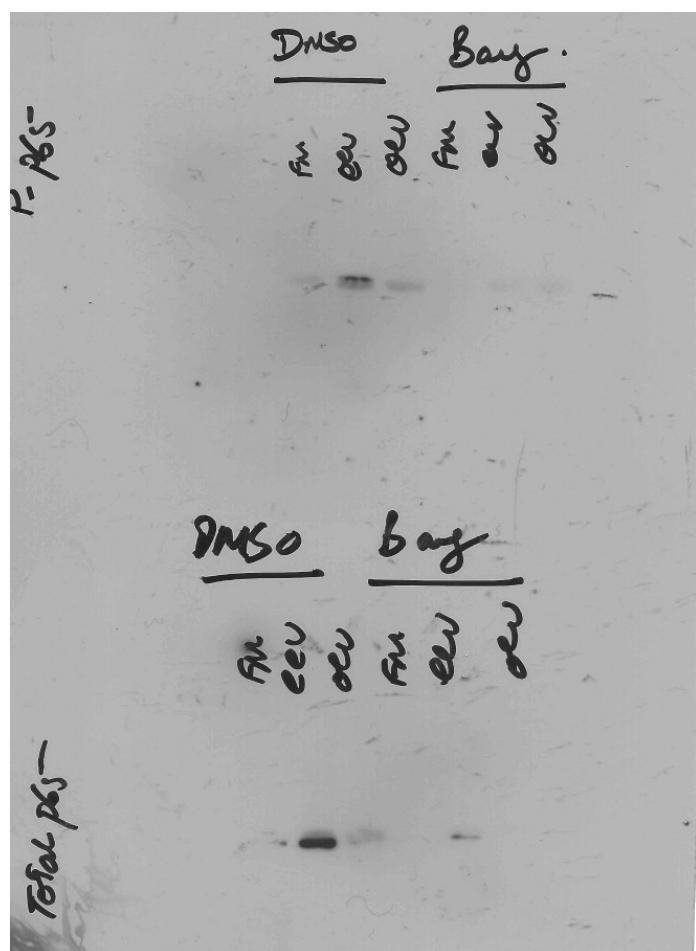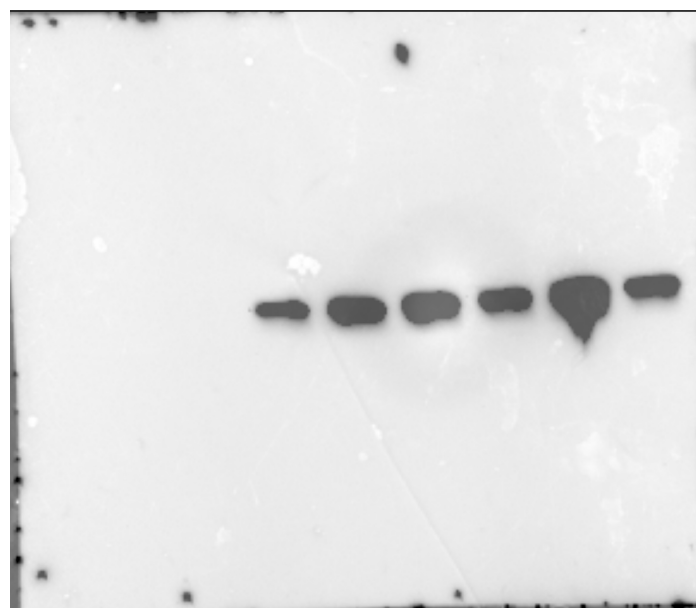

βACTIN

Supplement: Supplementary file 8 — Source Data [file 41467_2022_33235_MOESM8_ESM.zip › SOURCE DATA-92/figure 5l-source data.pdf]

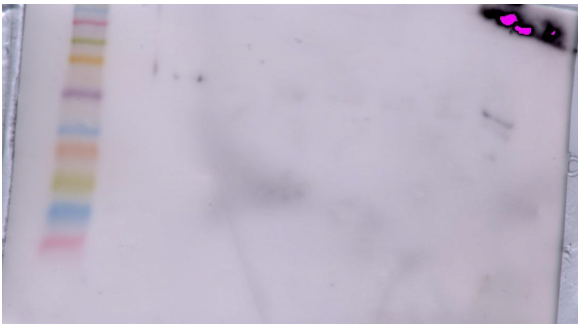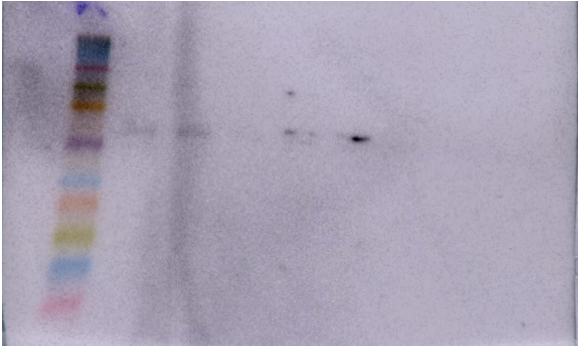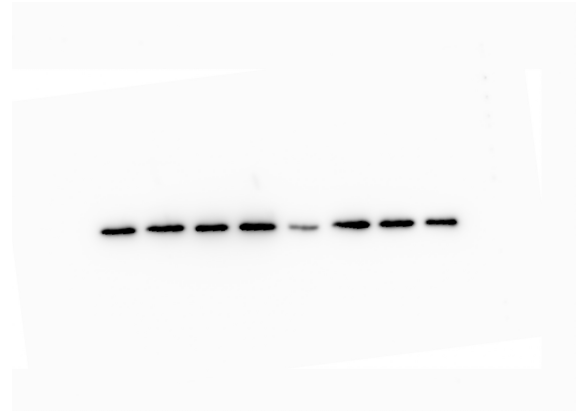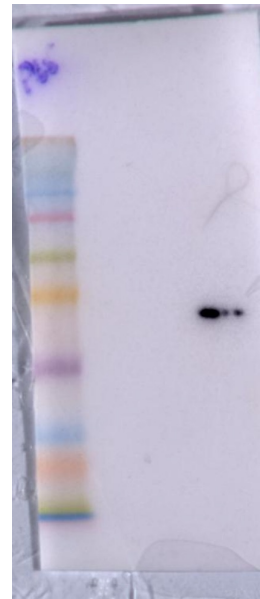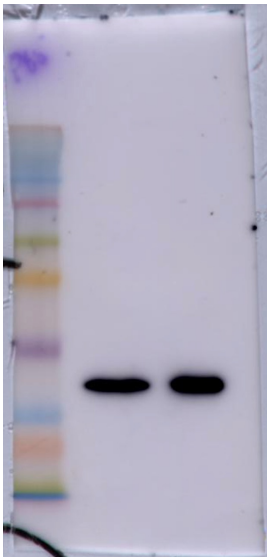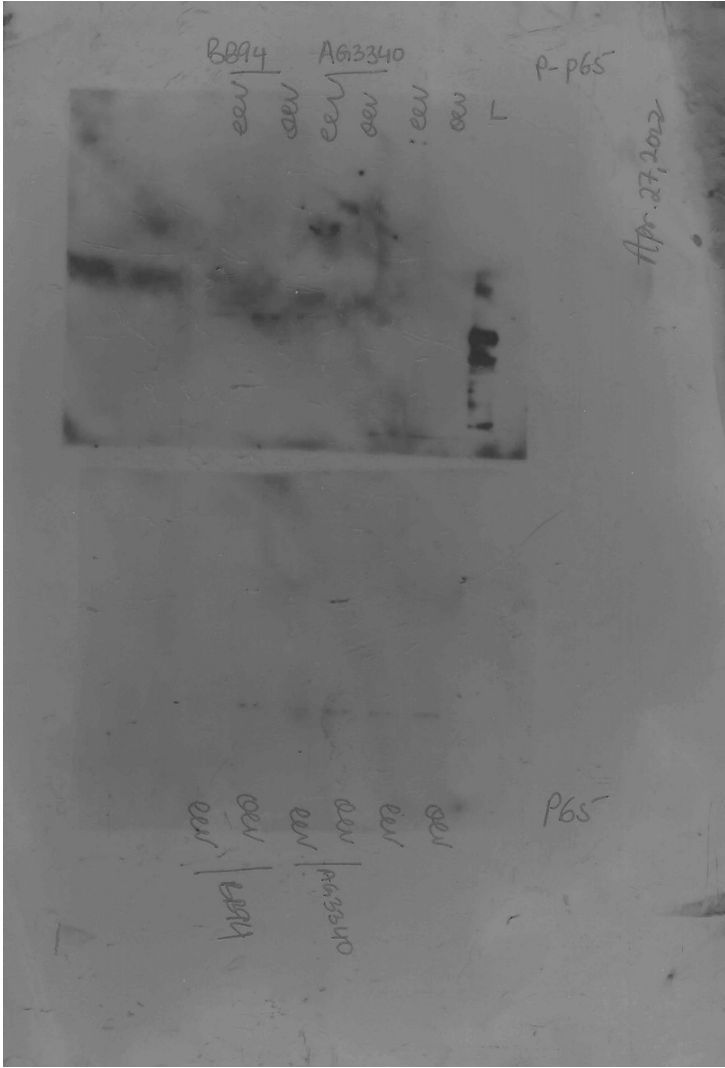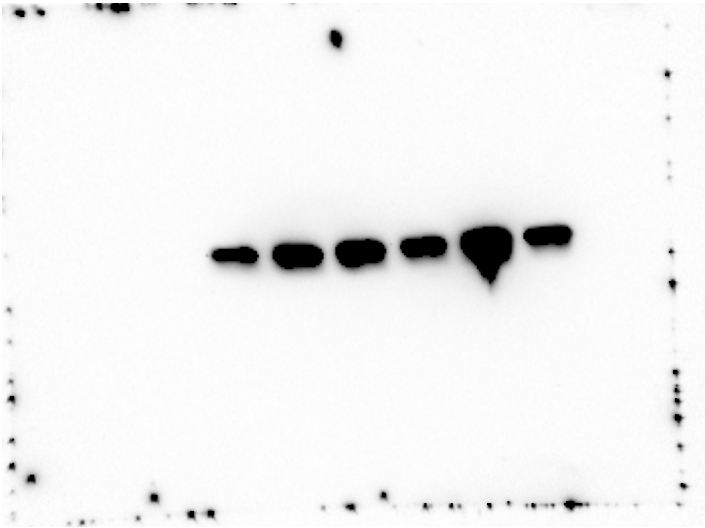

FIGURE 5m-source data

Supplement: Supplementary file 8 — Source Data [file 41467_2022_33235_MOESM8_ESM.zip › SOURCE DATA-92/figure 5m-source data.pdf]

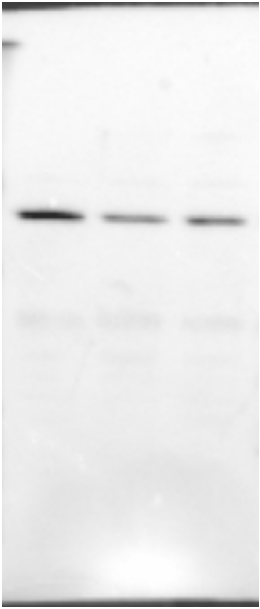

NCD

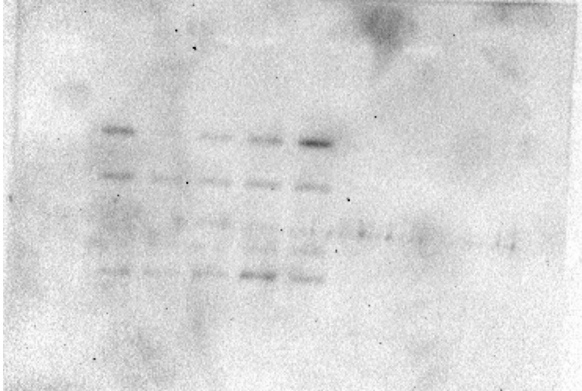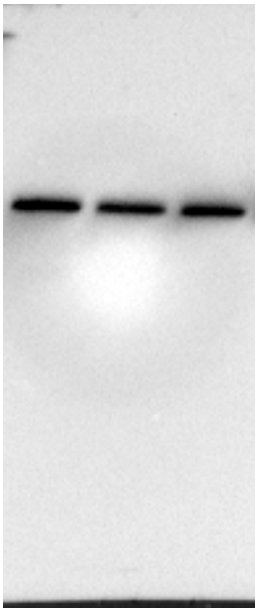

$\beta$ ACTIN

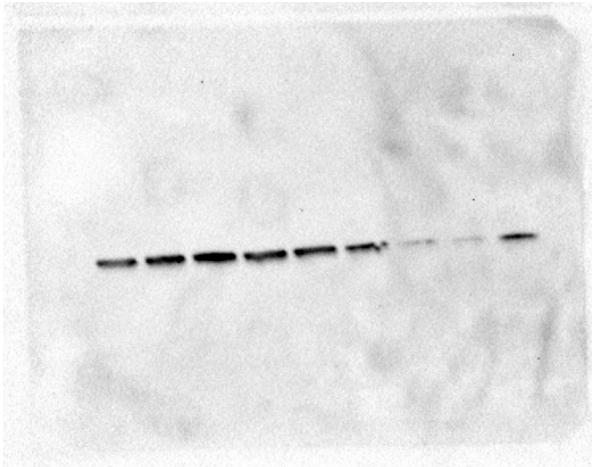

Supplement: Supplementary file 8 — Source Data [file 41467_2022_33235_MOESM8_ESM.zip › SOURCE DATA-92/figure 5q-source data.pdf]

(a)

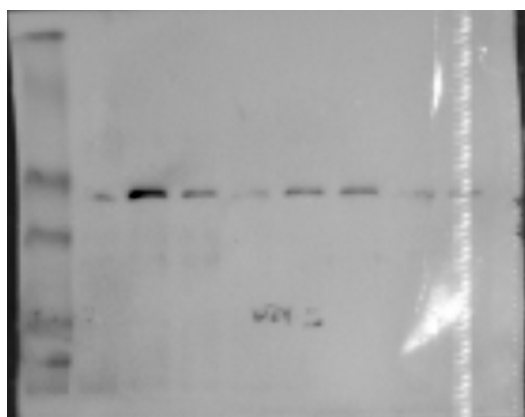

NICD

(b)

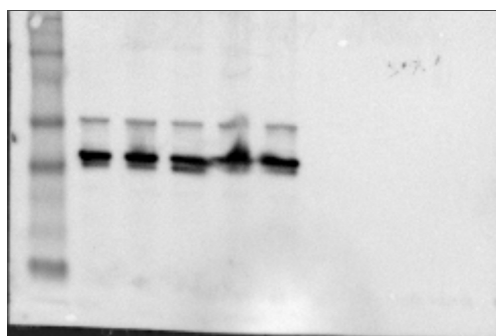

NOTCH1

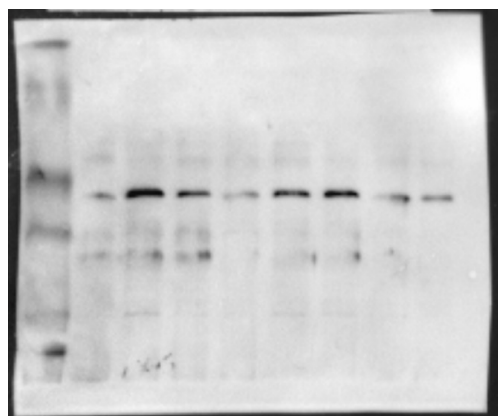

SOX2

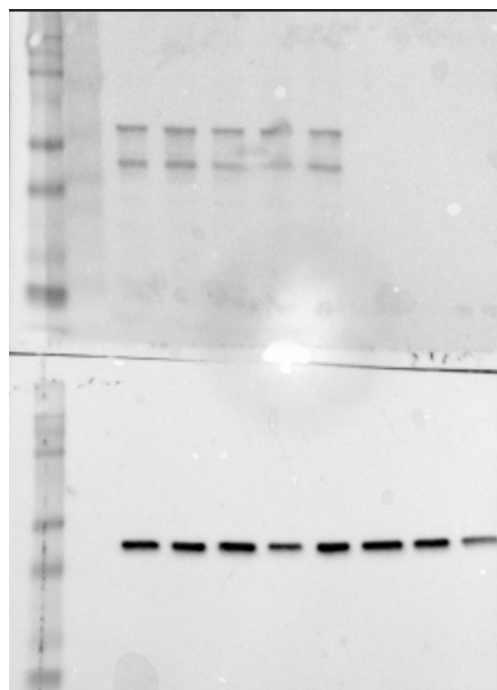

VIM

bACTIN

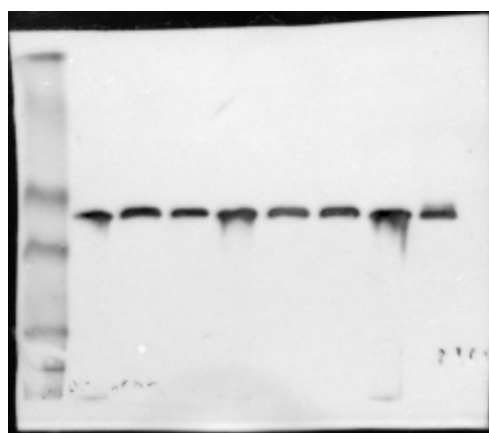

bACTIN

Supplement: Supplementary file 8 — Source Data [file 41467_2022_33235_MOESM8_ESM.zip › SOURCE DATA-92/suppl fig 10ab-source data.pdf]

|     |   |   |   |   | AG3340 |   | BB94 |   |
|-----|---|---|---|---|--------|---|------|---|
| SM  | + | + | + | + | +      | + | +    | + |
| OEV | - | + | - | - | +      | - | +    | - |
| EEV | - | - | + | + | -      | + | -    | + |

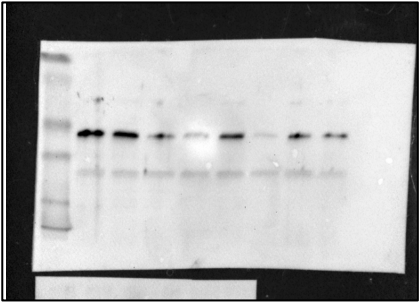

NICD

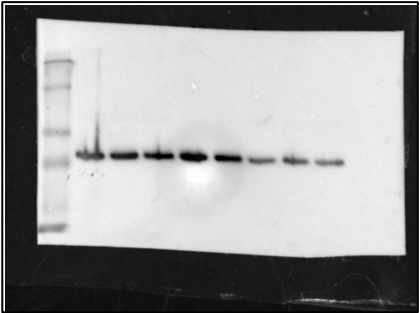

B ACTIN

Supplement: Supplementary file 8 — Source Data [file 41467_2022_33235_MOESM8_ESM.zip › SOURCE DATA-92/suppl fig 10e-source data.pdf]

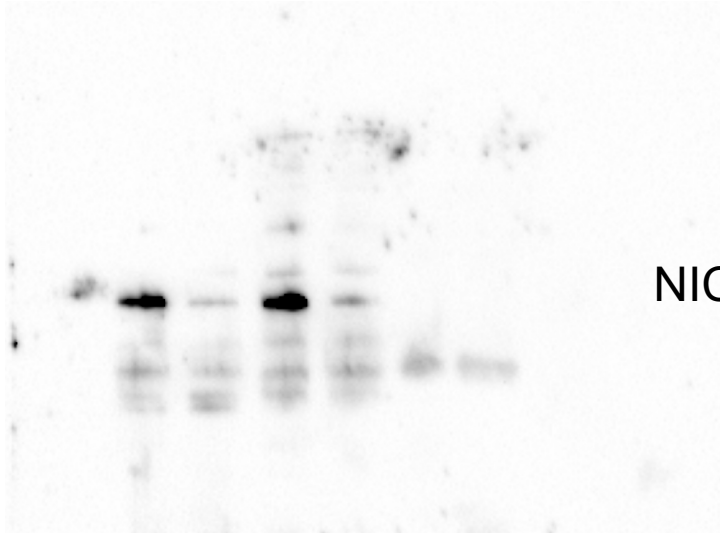

NICD

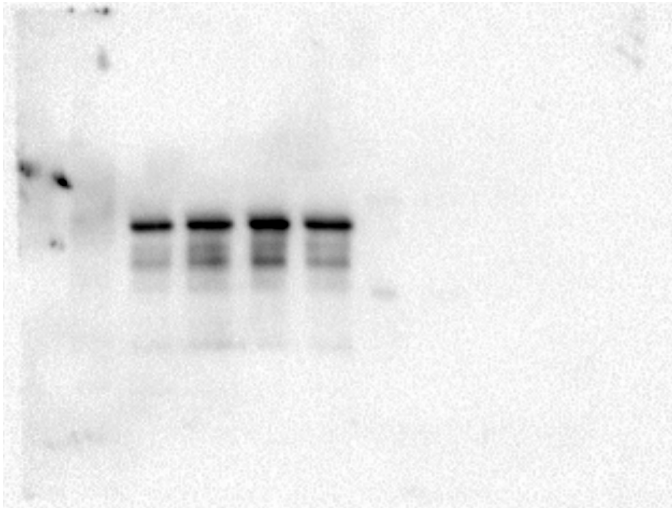

VIM

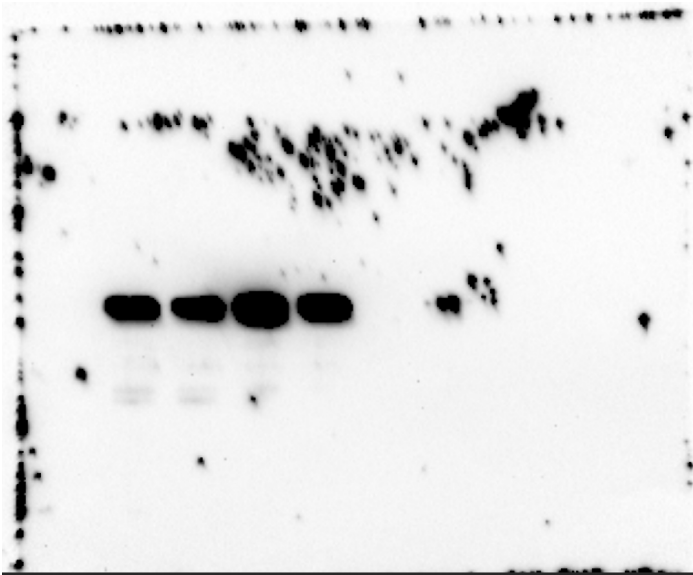

bACTIN

Supplement: Supplementary file 8 — Source Data [file 41467_2022_33235_MOESM8_ESM.zip › SOURCE DATA-92/suppl fig 11b-source data.pdf]
